# Ethanol extract of propolis relieves exercise-induced fatigue via modulating the metabolites and gut microbiota in mice

**DOI:** 10.3389/fnut.2025.1549913

**Published:** 2025-03-26

**Authors:** Shan Huang, Xiaofei Yang, Jingxuan Ma, Chen Li, Yajing Wang, Zhaoxia Wu

**Affiliations:** ^1^College of Food Science, Shenyang Agricultural University, Shenyang, Liaoning, China; ^2^College of Grain Science and Technology, Shenyang Normal University, Shenyang, Liaoning, China; ^3^College of Food and Health, Jinzhou Medical University, Jinzhou, Liaoning, China

**Keywords:** propolis, ethanol extract of propolis, exercise-induced fatigue, metabolomics, gut microbiota

## Abstract

**Background:**

Propolis, a natural mixture rich in bioactive compounds, has shown the potential to relieve exercise-induced fatigue. However, the underlying mechanism remains unclear. This study aimed to explore the anti-fatigue effects of ethanol extract of propolis (EEP) and its potential mechanisms.

**Methods:**

Male C57BL/6 mice aged 6–8 weeks were subjected to swim training with or without EEP supplementation (400 mg/kg.bw) for 3 weeks, followed by a exhaustive swimming test to simulate exercise-induced fatigue. The exhaustion time and fatigue-related biochemical indices were measured to assess the anti-fatigue effects. The anti-fatigue mechanism of EEP was further investigated using untargeted serum metabolomics and 16S rRNA gene sequencing of the gut microbiota.

**Results:**

The results showed that supplementation with EEP significantly increased the exhaustive swimming time of the mice by 27.64%, with no significant effects on body weight, food intake, or viscera and muscle index among the 3 groups. Biochemical analysis indicated that EEP effectively alleviated fatigue-related biochemical indices caused by excessive exercise, including liver glycogen (LG), muscle glycogen (MG), blood lactate (BLA), blood urea nitrogen (BUN), lactate dehydrogenase (LDH), interleukin-6 (IL-6), interleukin-1β (IL-1β), tumor necrosis factor-alpha (TNF-*α*), superoxide dismutase (SOD), total antioxidant capacity (T-AOC), glutathione peroxidase (GSH-Px), and malondialdehyde (MDA). Serum metabolomics analysis revealed that EEP reversed the levels of 6 key metabolites (Gamma-Aminobutyric acid, pipecolic acid, L-isoleucine, sucrose, succinic acid, and L-carnitine), which are involved in 7 metabolic pathways related to energy metabolism, amino acid metabolism, and carbohydrate metabolism. 16S rRNA sequencing analysis of the cecal contents showed that EEP altered the composition and structure of the gut microbiota, increasing the abundance of butyrate-producing bacteria and reducing the abundance of harmful bacteria. Correlation analysis revealed that specific bacterial genera were closely related to certain differential metabolites and biochemical indices.

**Conclusion:**

Our study showed that EEP significantly increased exercise endurance in mice and exerted anti-fatigue effects by modulating key metabolites and the gut microbiota.

## 1 Introduction

Exercise-induced fatigue refers to a physiological state that occurs after sustained or intense physical activity, and is characterized by a decline in muscle strength and athletic performance (1, 2). Excessive exercise can adversely affect the performance and work efficiency of athletes, sports enthusiasts, and physical laborers (3). Without timely intervention, this may lead to serious secondary problems and threaten health (4). Therefore, effective prevention and alleviation of exercise-induced fatigue has become a topic of extensive research.

In recent years, there has been great interest in the anti-fatigue effects of traditional drugs and natural bioactive compounds, which have fewer side effects and better biocompatibility compared to synthetic drugs (5–7). Propolis is a natural viscous substance made by bees collecting saps, resins, and mucilages from different parts of plants and then mixing them with beeswax and several bee enzymes (8). While propolis composition varies according to geographical location and botanical origin, polyphenols, including flavonoids and phenolic acids, represent the primary active ingredients (9). Recently, propolis has been widely incorporated into diverse healthcare products due to its antibacterial, antioxidant, immunomodulatory, and anti-inflammatory properties (10–13).

Quercetin, a flavonoid found in propolis, is known to benefit muscle health. Dietary supplementation with quercetin increases antioxidant capacity and muscle mitochondrial fatty acid *β*-oxidation while mitigating muscle damage (14, 15). Reactive oxygen species (ROS) derived from muscles contribute to muscle fatigue, and propolis has been shown to reduce ROS in isolated cardiac mitochondria (16, 17). Propolis inhibits the accumulation of advanced glycation end products (AGEs) in skeletal muscles, thereby preserving muscle function and promoting post-exercise recovery (18, 19). Propolis supplementation has been shown to have beneficial effects on oxidative stress and inflammation following intense physical activity in healthy male subjects, and to enhance muscle fatigue recovery by alleviating central fatigue (20, 21). However, although existing studies have indicated the potential of propolis to combat fatigue, most have focused on individual mechanisms, such as antioxidant, anti-inflammatory, and muscle protection effects, accordingly lacking a comprehensive understanding of its overall anti-fatigue mechanisms.

Research has demonstrated that the occurrence and development of exercise-induced fatigue are closely related to the excessive accumulation of lactic acid, decrease in energy, oxidative stress, and inflammatory responses, which are directly related to the imbalance of the gut microbiota, and this imbalance can aggravate the symptoms of fatigue (22, 23). Therefore, the change in gut microbiota is not only related to the occurrence and development of fatigue but may also be one of the key mechanisms by which propolis exerts anti-fatigue effects. Metabolomics exhibit higher sensitivity and specificity compared to traditional detection methods, enabling accurate detection of subtle metabolic changes (24–26). Combined application of these technologies can potentially provide a more comprehensive understanding, thereby accelerating the discovery of anti-fatigue biomarkers and therapeutic targets for propolis.

**Figure 1:**
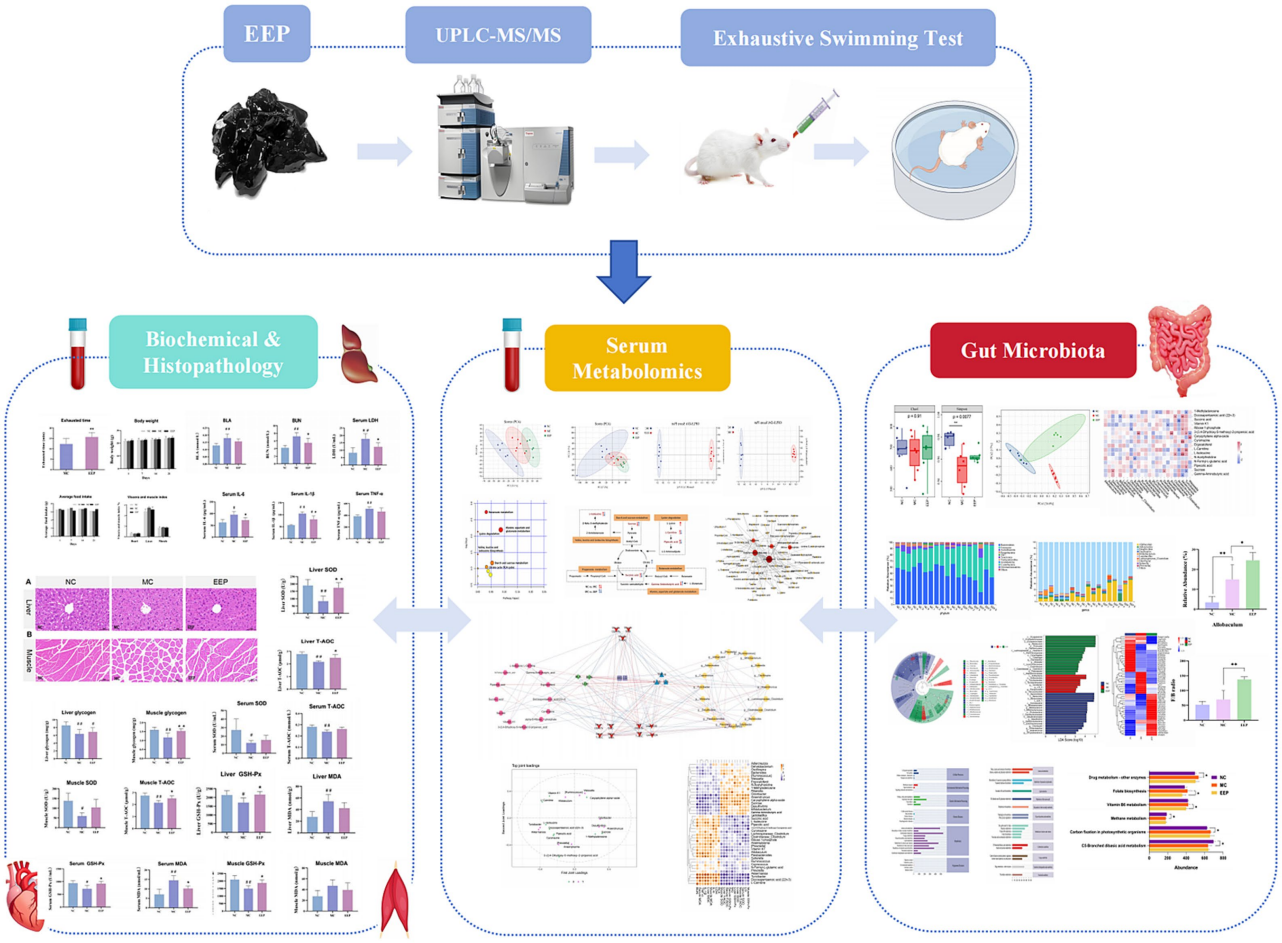
Flow chart of the research process.

This study used an exhaustive swimming mouse model to simulate exercise-induced fatigue, assessing the anti-fatigue effects of EEP by measuring exhaustive swimming time and fatigue-related biochemical markers. Untargeted metabolomics and 16S rRNA gene sequencing were employed to analyze the effects of EEP on the serum metabolite profile and gut microbiota of mice. Figure 1 depicts the flow chart of the research process.

## 2 Materials and methods

### 2.1 Chemical compositions analysis of ethanol extracts of propolis

**Figure 2:**
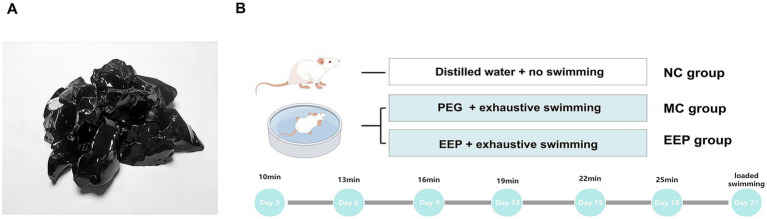
**(A)** Ethanol extract of propolis (EEP). **(B)** The scheme of animal experiments. Created with FigDraw.com.

EEP (Figure 2A) used in this study were obtained from Hangzhou Tianchu Miyuan Health Care Products Co., Ltd. (Hangzhou, China). This is an ethanolic extract of raw propolis (purity 98%). Raw propolis was collected from Shandong, China, and its botanical origin was poplar (Populus spp.). To analyze the chemical constituents of EEP, ultra-performance liquid chromatography–tandem mass spectrometry (UPLC–MS/MS) was employed. The analytical conditions were as follows: an Agilent SB-C18 column (1.8 μm, 2.1 mm × 100 mm) was used for UPLC. The mobile phase comprised solvent A (pure water with 0.1% formic acid) and solvent B (acetonitrile with 0.1% formic acid). Detection was conducted at a wavelength of 270 nm with an injection volume of 10 μL.

### 2.2 Animal experimental design

Male C57BL/6 mice, aged 6–8 weeks and weighing 23–25 g, were obtained from Beijing Huafukang Biotechnology Co., Ltd. (Beijing, China). Prior to experimentation, the mice underwent a one-week acclimation period under controlled conditions (22–24°C, 45–55% humidity, and 12 h light/dark cycle) with ad libitum access to standard chow and water. Subsequently, the mice were randomly assigned to three groups (*n* = 10/group): (1) normal control (NC), (2) model control (MC), and (3) EEP group (EEP). EEP group received an EEP feed dose of 400 mg/kg.bw, determined through preliminary experiments. NC group received an equivalent volume of distilled water, and MC group received an equivalent volume of polyethylene glycol (EEP solvent to control for solvent-related effects). All treatments were administered via intragastric gavage once daily for 3 weeks. All animal procedures were approved by the Experimental Animal Commission of Shenyang Agricultural University (IACUC No. 2023090703) and followed the guidelines for the care and use of laboratory animals.

### 2.3 Exhaustive swimming test and sample collection

An exhaustive swimming test was used to simulate exercise-induced fatigue, with the experimental methods slightly modified based on previously described methods (Figure 2B) (27). For swimming training, the mice (all mice except the NC group) were placed in a plastic container (depth: 50 cm, diameter: 40 cm) as a swimming pool. The water depth was 20 cm, and the water temperature was maintained at 22–25°C. The swimming training lasted for 3 weeks. Adaptive swimming exercises were conducted every 3 days, with the first exercise on the third day lasting 10 min, each subsequent exercise was increased by 3 min. The final loaded swimming with a weight equivalent to 7% of their body weight. Exhaustive swimming time was recorded when the mice lost coordinated movement and failed to return to the water surface within 7 s. After exhaustive swimming test, whole blood was collected via eyeball enucleation. The heart, liver, skeletal muscle (quadriceps femoris), and colonic contents were harvested for further analyses.

### 2.4 Biochemical assessments

After collection, blood was allowed to stand for 30 min. The serum was then prepared from the blood sample by centrifugation at 3000 rpm at 4°C for 15 min. Skeletal muscles and main organs were immediately harvested, rinsed, blotted dry, and weighed. IL-1β, IL-6, TNF-*α*, BUN, BLA, LDH, MDA, T-AOC, GSH-Px, SOD, LG and MG were determined according to the kit operation method (Ruixin Biological Technology Co., LTD, Quanzhou, China).

### 2.5 Histopathology analysis of the liver and skeletal muscle

Liver and skeletal muscle tissues were fixed in 10% paraformaldehyde, then processed and embedded in paraffin. Sections of 4 μm thickness were prepared using a microtome (RM2016, Leica Instrument Shanghai Ltd., China). The tissue sections were stained with hematoxylin and eosin (H&E), using a staining kit (G1120, Solarbio Sciences & Technology Co., Ltd., Beijing, China) and mounted with neutral gum. Microscopic images were captured using a Nikon imaging system (DS-U3, Nikon, Tokyo, Japan).

### 2.6 Untargeted metabolomics analysis

Serum samples (100 μL) were mixed with 400 μL of methanol, vortexed, and centrifuged at 12,000 rpm for 10 min at 4°C. The supernatant was evaporated under nitrogen and reconstituted in 150 μL of 2-chloro-L-phenylalanine (4 ppm in 80% methanol/20% water), then filtered for LC–MS analysis. Quality control (QC) samples monitored analytical variability.

Liquid chromatography was performed using a Vanquish UHPLC system with an HSS T3 column at 40°C. The flow rate was 0.3 mL/min, injection volume 2 μL. Positive and negative ion modes used gradient elution with formic acid, acetonitrile, and ammonium formate. Mass spectrometry detection used a Q Exactive mass spectrometer in MS1 and MS/MS scans.

Data processing was conducted using the R package ropls on the GenesCloud platform.^1^ Differential metabolites were identified by referencing the Human Metabolome Database (HMDB).^2^ Pathway enrichment and metabolite interaction analyses were performed using MetaboAnalyst 6.0.^3^

### 2.7 Gut microbiota analysis of cecal contents

Cecal contents collected on day 21 were frozen at −80°C for DNA extraction using a Soil DNA Kit. DNA concentration and purity were assessed via NanoDrop spectrophotometry and agarose gel electrophoresis.

The V3–V4 region of bacterial 16S rRNA genes was amplified using barcoded primers 338F and 806R. PCR conditions included initial denaturation, 25 cycles of denaturation, annealing, and extension, followed by final extension. PCR products were purified with VAHTS™ DNA Clean Beads and quantified using PicoGreen. Sequencing was performed on an Illumina NovaSeq platform.

Sequence data underwent demultiplexing and primer trimming (Demux and Cutadapt), followed by quality filtering, denoising, merging, and chimera removal (DADA2). Diversity analyses were conducted using QIIME2 and R (see text footnote 1), with microbial diversity comparisons using linear discriminate analysis (LDA) effect size (LEfSe). The functional potential of the gut microbiota was predicted using the PICRUSt2 bioinformatics tool.

### 2.8 Statistical analysis

Data were visualized using the GraphPad Prism software (version 9.5.1 GraphPad Software, Inc., La Jolla, CA, United States). The statistical significance of the data was analyzed by one-way ANOVA, followed by Bonferroni post-hoc comparison using IBM SPSS software (version 25.0 SPSS Inc., Chicago, IL, United States). *p* < 0.05 was significant and *p* < 0.01 was deemed to be extremely significant.

## 3 Results

### 3.1 Determination of the chemical composition of EEP

The analyzable total ion chromatogram (Supplementary Figure S1) was exported using Analyst 1.6.3. The spectral peaks were identified and characterized using the Metware Database, and 1942 substances were identified in the EEP. The results showed that polyphenols accounted for 68.34%, of which flavonoids accounted for 50.06%. In addition to flavonoids, other polyphenols such as phenolic acids accounted for 16.15%, lignans and coumarins accounted for 1.94%, and tannins accounted for 0.19%. Lipids accounted for 10.17%, and all other compounds accounted for 21.49%. The quantity of the compounds was calculated according to the area normalization method and ranked by peak area. The 100 most abundant compounds are listed in Supplementary Table S1.

### 3.2 Effects of EEP on the body weight, food intake, viscera and muscle index and endurance performance of mice

**Figure 3:**
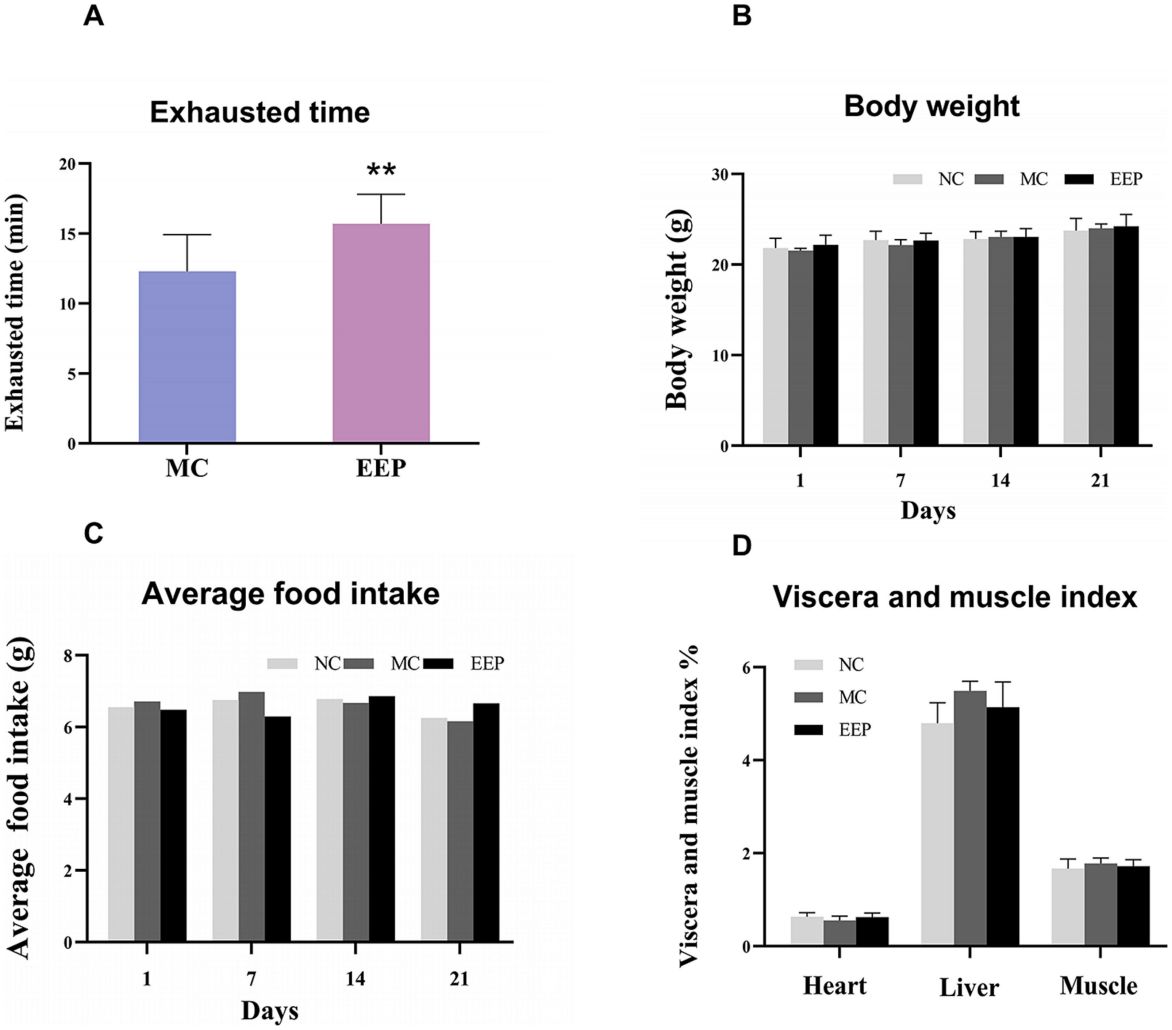
**(A)** Effects of EEP on exhausted time, **(B)** body weight, **(C)** average food intake, **(D)** viscera and muscle index (including heart, liver, muscle). Data are expressed as mean ± SD (*n* = 10), # *p* < 0.05 and ## *p* < 0.01 compared with NC, * *p* < 0.05 and ** *p* < 0.01 compared with MC.

We used an exhaustive swimming test to measure the endurance performance of the mice, and the results showed in Figure 3A. Compared with the MC group, the swimming time of the EEP group was significantly increased by 27.64%. There were no significant differences in body weight, food intake, and the viscera and muscle index among the groups (Figures 3B–D). This suggested that EEP may have had a positive effect on exercise endurance in mice without negative effects on physiological indices and was safe and non-toxic.

### 3.3 Effects of EEP on liver glycogen and muscle glycogen

**Figure 4:**
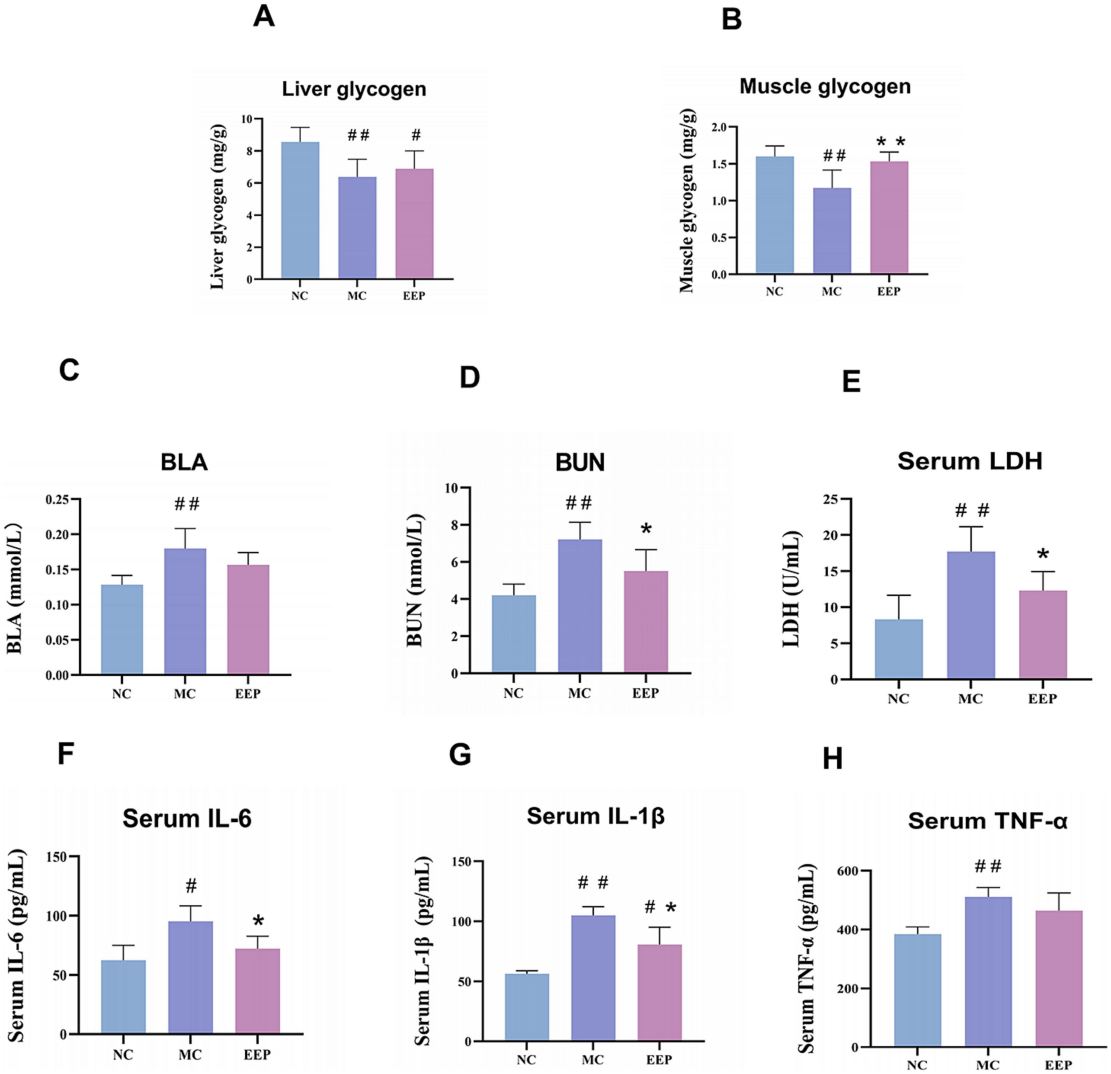
Effects of EEP on **(A)** liver glycogen (LG), **(B)** muscle glycogen (MG), **(C)** blood lactate (BLA), **(D)** blood urea nitrogen (BUN), **(E)** serum lactate dehydrogenase (LDH), **(F)** serum interleukin 6 (IL-6), **(G)** serum interleukin 1 beta (IL-1β), **(H)** serum tumor necrosis factor-alpha (TNF-*α*) of mice. Data are expressed as mean ± SD (*n* = 6), # *p* < 0.05 and ## *p* < 0.01 compared with NC, * *p* < 0.05 and ** *p* < 0.01 compared with MC.

Glycogen reduction is an important contributor to fatigue, especially during prolonged high-intensity exercise. In this study, EEP relieved the decrease in LG and MG levels caused by exercise-induced fatigue (Figures 4A,B). The LG and MG of mice after exercise were decreased by 34.38% (*p* < 0.01) and 36.75% (*p* < 0.01) compared to the NC group. The reduction of LG and MG by exercise-induced fatigue was improved after EEP supplementation, which increased by 8.16 and 30.77% (*p* < 0.01), respectively. These data indicate that EEP supplementation alleviated exercise-induced loss of LG and MG in mice.

### 3.4 Effects of EEP on metabolite accumulation

Fatigue is often accompanied by muscle cell damage and metabolite accumulation, as shown in Figures 4C–E. Compared to the NC group, the levels of BLA, BUN, and LDH in the MC group were significantly increased. After EEP supplementation, these levels decreased. Specifically, the levels of BLA, BUN, and LDH in the MC group increased by 42.34% (*p* < 0.01), 71.71% (*p* < 0.01), and 113.05% (*p* < 0.01), respectively, compared to the NC group. In contrast, the EEP group showed a reduction in BLA, BUN, and LDH by 17.14% (*p* > 0.05), 30.98% (*p* < 0.05), and 43.76% (*p* < 0.05), respectively, compared to the MC group. The capacity of EEP to mitigate metabolite accumulation could be associated with its potential anti-fatigue effects.

### 3.5 Effect of EEP on reduce inflammation

Excessive exercise often leads to an inflammatory response, as shown in Figures 4F–H, the levels of inflammatory factors in mice in the MC group were significantly increased, but decreased after EEP supplementation. The IL-6, IL-1β and TNF-*α* of the MC group were increased by 52.4% (*p* < 0.05), 86.16% (*p* < 0.01), and 32.85% (*p* < 0.01) compared to the NC group, respectively. The IL-6, IL-1β, and TNF-α of the EEP group were reduced by 24.29% (*p* < 0.05), 23.22% (*p* < 0.05), and 9.15% (*p* > 0.05) compared to the MC group, respectively. These results suggest that EEP can alleviate the inflammatory response induced by excessive exercise.

### 3.6 Effect of EEP on oxidative stress

**Figure 5:**
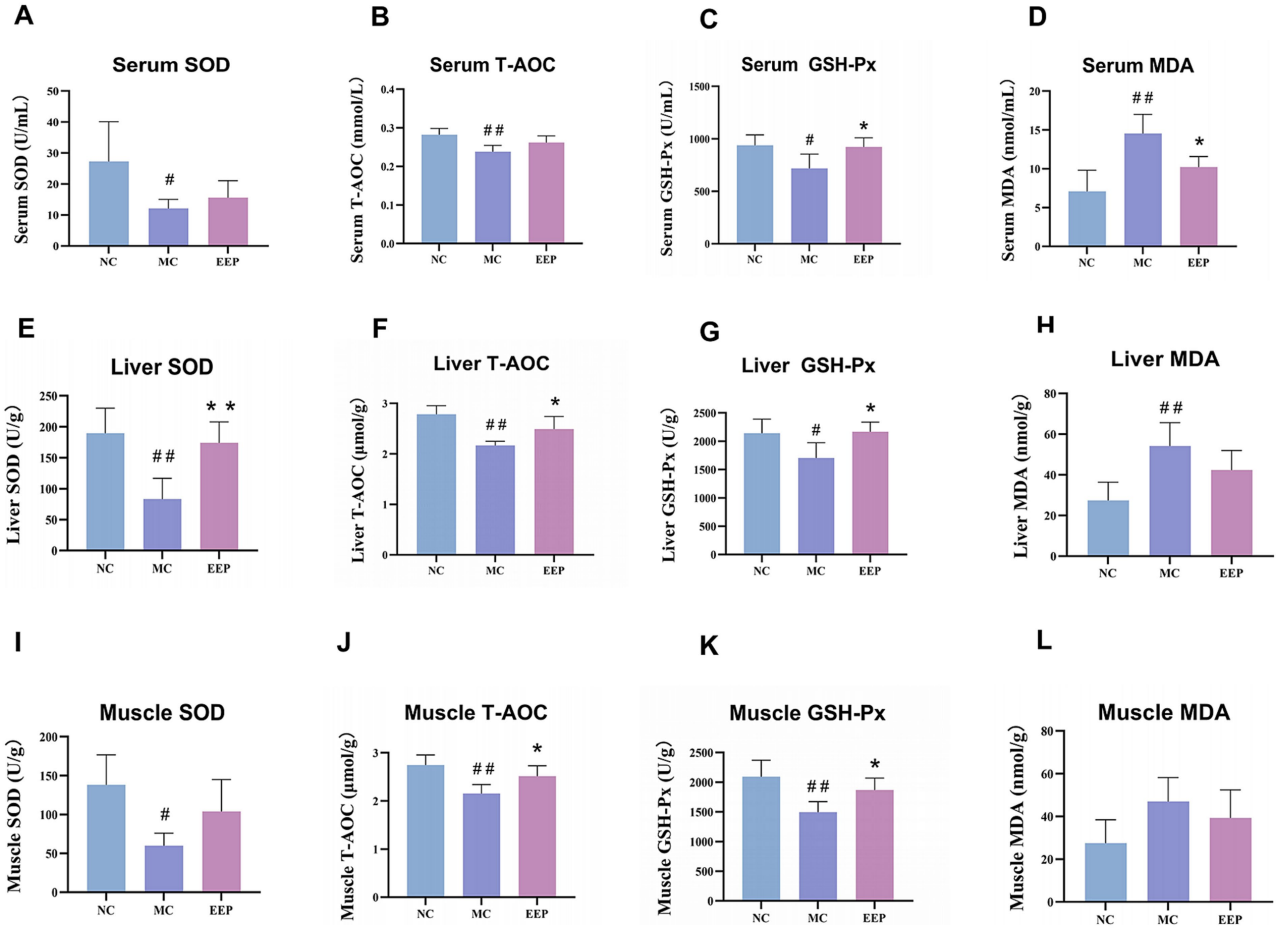
Effects of EEP on **(A)** serum superoxide dismutase (SOD), **(B)** serum total antioxidant capacity (T-AOC), **(C)** serum glutathione peroxidase (GSH-Px), **(D)** serum malondialdehyde (MDA), **(E)** liver SOD, **(F)** liver T-AOC, **(G)** liver GSH-Px, **(H)** liver MDA, **(I)** muscle SOD, **(J)** muscle T-AOC, **(K)** muscle GSH-Px, and **(L)** muscle MDA of mice. Data are expressed as mean ± SD (*n* = 6), # *p* < 0.05 and ## *p* < 0.01 compared with NC, * *p* < 0.05 and ** *p* < 0.01 compared with MC.

During intense exercise, the body produces more free radicals, leading to increased stress on the antioxidant defense system. As shown in Figures 5A–L, the antioxidant capacities of the serum, liver, and skeletal muscle in the MC group were significantly decreased, when compared to the NC group. EEP reversed the above trend. In the EEP group, the SOD levels in serum, liver, and skeletal muscle increased by 28.9% (*p* > 0.05), 109.15% (*p* < 0.01), and 73.78% (*p* > 0.05), respectively. Meanwhile, the T-AOC of the serum, liver, and skeletal muscle increased by 8.83% (*p* > 0.05), 16.83% (*p* < 0.05) and 14.60% (*p* < 0.05) respectively. Lastly, the GSH-Px of the serum, liver, and skeletal muscle significantly increased by 26.21% (*p* < 0.05), 25.83% (*p* < 0.05) and 22.83% (*p* < 0.05), respectively. To the contrary, the MDA in the serum, liver, and skeletal muscle decreased by 29.84% (*p* < 0.05), 21.74% (*p* > 0.05), and 16.32% (*p* > 0.05), respectively. These results indicate that EEP ameliorated oxidative stress in mice, thereby delaying fatigue.

### 3.7 Effects of EEP on liver and skeletal muscle histopathology

**Figure 6:**
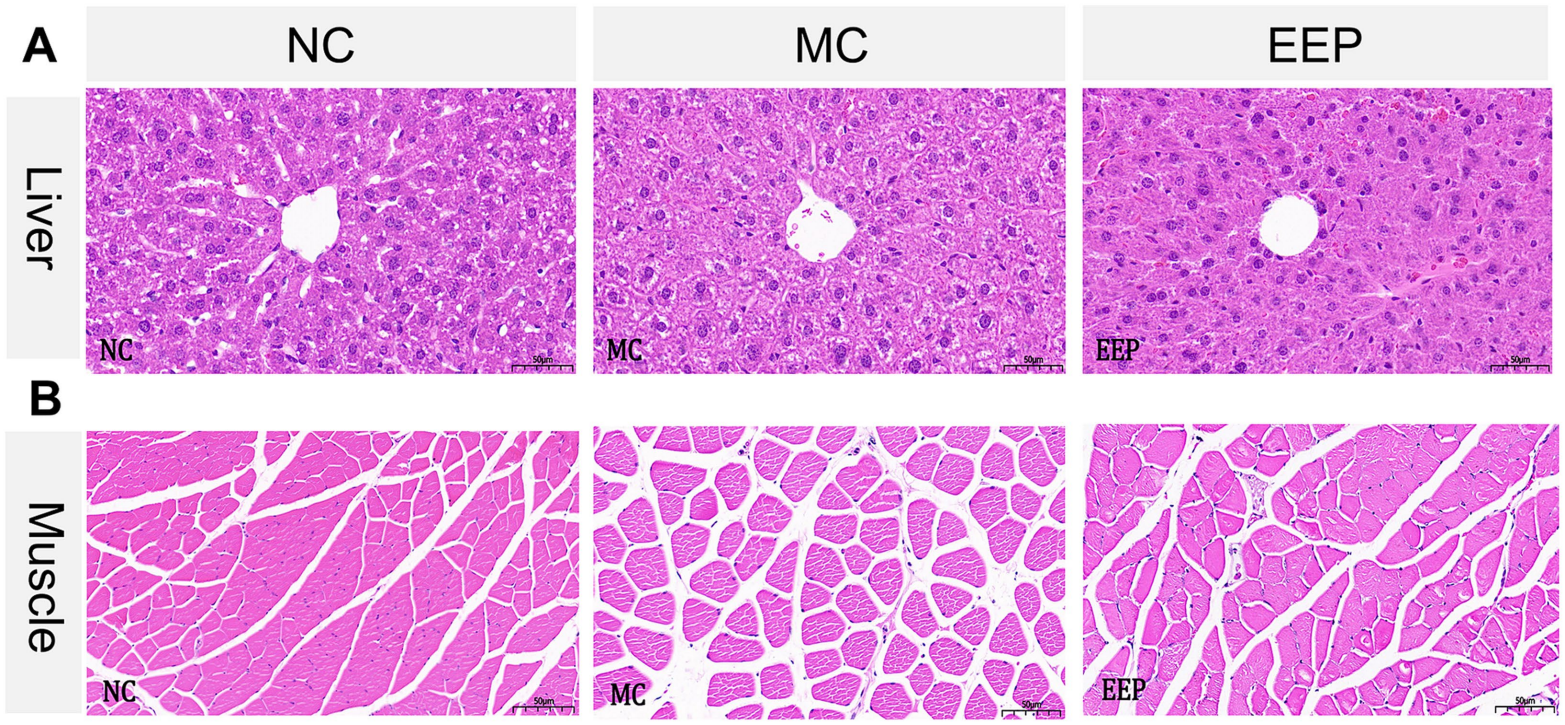
Representative histopathology sections of the **(A)** liver and **(B)** skeletal muscle of mice (magnification 20×, scale bar 50 μm).

H&E stained liver tissue sections from the NC group showed hepatocyte cords arranged orderly, with uniform and evenly distributed nuclei. No significant inflammatory cell infiltration or necrotic lesions were observed in the sample. Liver tissue architecture in both MC and EEP groups did not show obvious abnormal changes compared to that in the control group, indicating that EEP supplementation did not induce hepatic toxicity in mice (Figure 6A).

In H&E stained muscle tissue sections, compared with the NC group, some muscle fibers in the MC group showed a loosened structure, gaps, increased interfiber spacing, and slight degeneration. However, the EEP group showed a reduction in muscle fiber gaps and structural loosening. Muscle fibers in the EEP group were more densely packed, and the overall morphology appeared more intact (Figure 6B). These results indicated that EEP supplementation partially repaired or alleviated muscle fiber damage induced by exercise fatigue, maintaining normal muscle structure and function.

### 3.8 Effects of EEP on the metabolic profiles of mice

**Figure 7:**
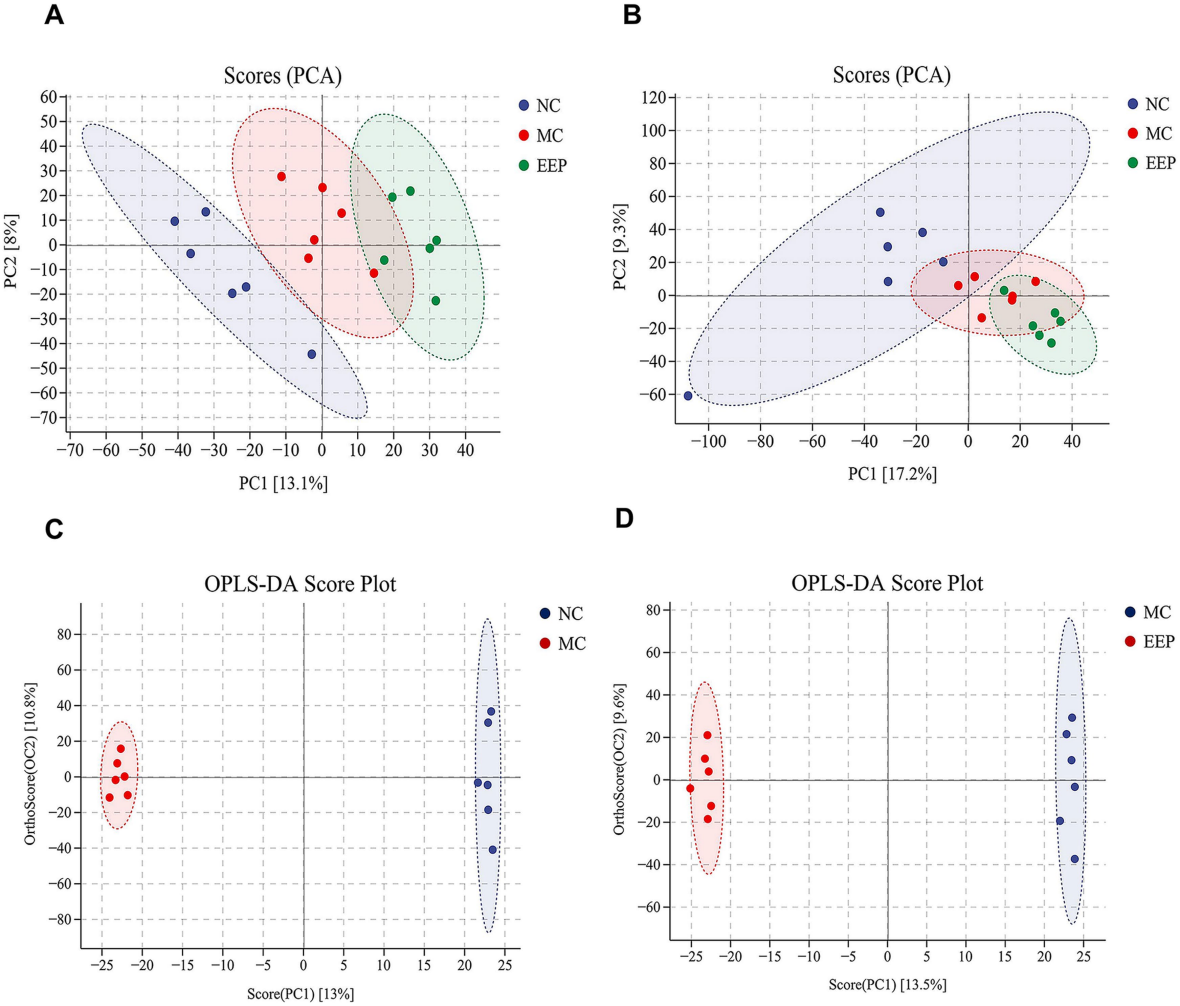
**(A)** Principal component analysis (PCA) of positive ion (NC vs. MC vs. EEP). **(B)** PCA of negative ion (NC vs. MC vs. EEP). **(C)** Orthogonal partial least squares-discriminant analysis (OPLS-DA) score plot (NC vs. MC). **(D)** OPLS-DA score plot (MC vs. EEP). *n* = 6 for each group.

To elucidate the anti-fatigue effects of EEP, untargeted metabolomics was conducted to identify the serum metabolites in mice. The results of principal component analysis (PCA) under both positive (Figure 7A) and negative (Figure 7B) ion scanning modes showed that all samples exhibited significant clustering, particularly in the positive ion mode. To better distinguish between the groups and improve the validity and analysis of the models, we used orthogonal partial least squares-discriminant analysis (OPLS-DA), which can easily be divided into two distinct clusters after pairwise comparison, as shown in Figures 7C,D.

In OPLS-DA, variables important for projection (VIP) and fold change (FC) were used to identify biologically significant differential metabolites. In this study, VIP >1 and FC >1.2 were used as the screening criteria. Compared to the NC group, 111 serum metabolites (71 upregulated and 40 downregulated) were screened in the MC group, and 95 serum metabolites (35 upregulated and 60 downregulated) were screened in the EEP group (Supplementary Tables S2, S3). Among these, 22 metabolites were found to be associated with EEP relieving exercise-induced fatigue. The chemical structures of these metabolites were further identified and verified using the HMDB, ultimately confirming 16 differential metabolites, as shown in Table 1.

**Table 1:** Metabolites regulated by intense exercise and EEP.

Compounds	M/Z	RT(s)	Ion	Chemical formula	NC vs. MC	MC vs. EEP
Succinic acid	117.0549	58.6	[M − H]−	C_4_H_6_O_4_	Up	Down
Pipecolic acid	130.0502	73.8	[M + H]+	C_6_H_11_NO_2_	Up	Down
L-Isoleucine	132.0533	318.2	[M + H]+	C_6_H_13_NO_2_	Up	Down
L-Carnitine	162.0565	239	[M + H]+	C_7_H_15_NO_3_	Up	Down
Cyromazine	166.0982	670.9	[M]+	C_6_H_10_N_6_	Up	Down
N-Formyl-L-glutamic acid	176.0716	333.3	[M + H]+	C_6_H_9_NO_5_	Up	Down
3-(3,4-Dihydroxy-5-methoxy)-2-propenoic acid	193.0493	512.6	[M + H − H_2_O]+	C_10_H_10_O_5_	Up	Down
Ribose 1-phosphate	229.0098	45.5	[M − H]−	C_5_H_11_O_8_P	Up	Down
Docosapentaenoic acid (22n-3)	329.2476	616.3	[M − H]−	C_22_H_34_O_2_	Up	Down
Vitamin K1	449.0934	566.1	[M − H]−	C_31_H_46_O_2_	Up	Down
Gamma-Aminobutyric acid	103.0551	318.3	[M]+	C_4_H_9_NO_2_	Down	Up
N-Acetylhistidine	198.0856	59.3	[M + H]+	C_8_H_11_N_3_O_3_	Down	Up
Caryophyllene alpha-oxide	221.1909	465.5	[M + H]+	C_15_H_24_O	Down	Up
1-Methyladenosine	281.1204	660.7	[M]−	C_11_H_15_N_5_O_4_	Down	Up
Sucrose	343.2981	408.7	[M + H]+	C_12_H_22_O_11_	Down	Up
Ergocalciferol	396.3444	480.4	[M]+	C_28_H_44_O	Down	Up

**Figure 8:**
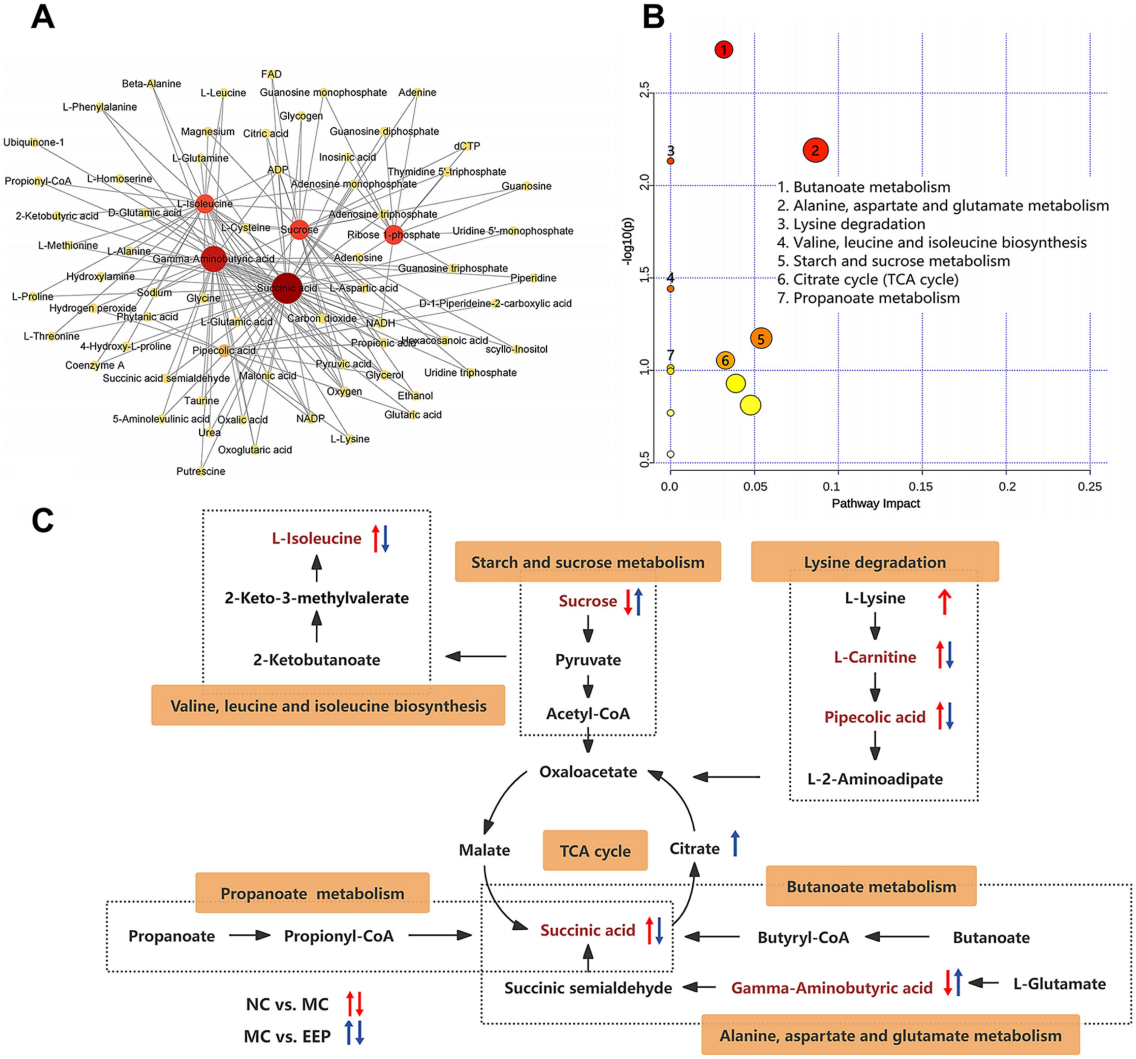
**(A)** Metabolite-metabolite interaction network. **(B)** KEGG pathway enrichment analysis of differential metabolites. **(C)** Correlation networks of differential metabolites effects on exercise-induced fatigue in mice.

Metabolite-metabolite interactions analysis can help highlight the potential functional relationships between the 16 differential metabolites in mice with EEP. Metabolite-metabolite interaction analysis was performed using the MetaboAnalyst website, and a metabolite-metabolite interaction network was mapped using Cytoscape with a degree greater than 2 (Figure 8A). Each metabolite node in the figure represents the degree from small to large and from shallow to deep. Succinic acid, Gamma-Aminobutyric acid (GABA), sucrose, ribose 1-phosphate, L-isoleucine, and pipecolic acid are all key nodes in the network.

The effects of EEP supplementation on metabolic pathways were analyzed using the Metaboanalyst 6.0 Online analysis platform. As shown in Figure 8B, in the positive and negative ion modes, the metabolic pathways significantly affected (−log10(P) > 1.0) by EEP included butanoate metabolism, alanine, aspartate and glutamate metabolism, lysine degradation, valine, leucine, and isoleucine biosynthesis, starch and sucrose metabolism, tricarboxylic acid (TCA) cycle, and propanoate metabolism.

A correlation network based on the Kyoto Encyclopedia of Genes and Genomes (KEGG) database was constructed to illustrate the potential links between the metabolic pathways (Figure 8C). The tricarboxylic acid cycle is the key node connecting amino acid metabolism (alanine, aspartate, and glutamate metabolism, lysine degradation, valine, leucine, and isoleucine biosynthesis), carbohydrate metabolism (starch and sucrose metabolism), fatty acid metabolism (butanoate metabolism and propanoate metabolism). These results suggest that EEP may alleviate exercise fatigue in mice by affecting these differential metabolites and metabolic pathways.

### 3.9 Effect of EEP on gut microbiota of mice

**Figure 9:**
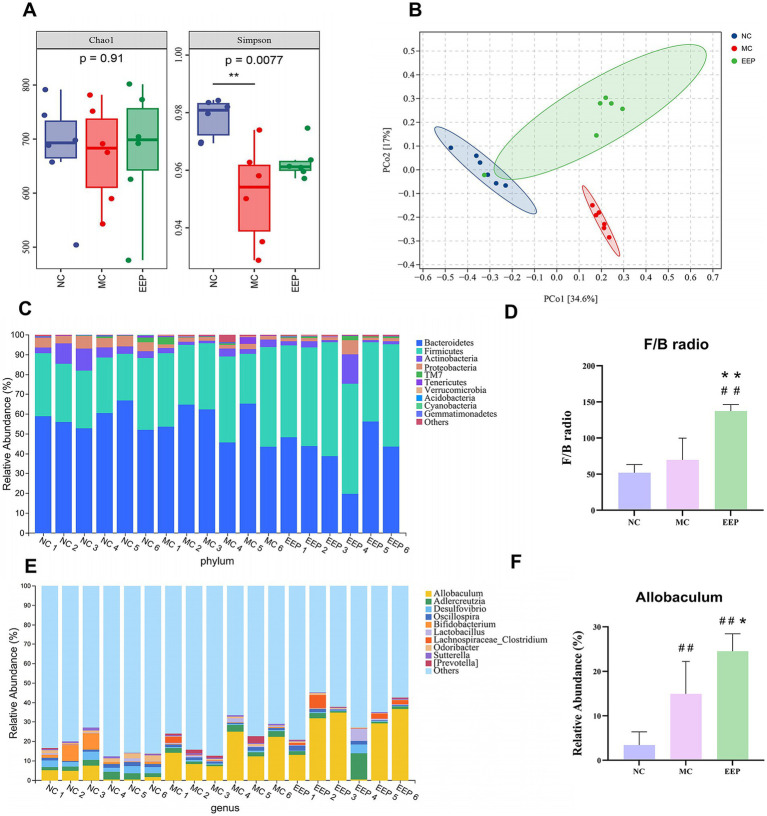
Effects of EEP intervention on the structure and function of cecal contents microbial community. **(A)** Alpha-diversity, richness index: Chao1, diversity index: Shannon. **(B)** Beta-diversity, principal coordinates analysis (PCoA) plot of gut microbiota based on weighted unifrac. **(C)** Relative abundance of gut microbiota at phylum level. **(D)** F/B radio. **(E)** Relative abundance of gut microbiota at genus level. **(F)** Relative abundance of *Allobaculum*. Data are expressed as mean ± SD (*n* = 6), # *p* < 0.05 and ## *p* < 0.01 compared with NC, * *p* < 0.05 and ** *p* < 0.01 compared with MC.

We performed 16S rRNA sequencing to explore the role of EEP in the regulation of the gut microbiota in mice. The Chao1 index was used to estimate species richness, and the Simpson index was used to measure species diversity (Figure 9A). Chao1 indicated no significant differences among all groups (*p* = 0.27), while the Simpson index revealed significant differences between the NC group and MC group (*p* = 0.0077). Principal coordinates analysis (PCoA) used to examine the differences between the 3 groups, and the results showed a clear separation of the groups (Figure 9B). These results indicated that both intense exercise and EEP supplementation altered the gut microbiota composition in mice, but the mechanisms were different. We further analyzed the differences in microbial community structure between different groups at the phylum and genus levels. The top 10 classifications with the highest abundances were selected, and Bacteroidetes, Firmicutes, Actinobacteria, and Proteobacteria were determined as the predominant phyla, accounting for more than 97%, but the composition was different among all groups (Figure 9C), the ratio of Firmicutes to Bacteroidetes in EEP group was the highest among the 3 groups (NC: 51.14%, MC: 65.59%, EEP:119.85%) (Figure 9D). The dominant genera were *Allobaculum*, *Adlercreutzia*, *Desulfovibrio*, and *Oscillospira* (Figure 9E), the EEP group had the highest proportion of *Allobaculum* abundance among all groups (NC: 3.42%, MC: 14.95%, EEP: 24.38%) (Figure 9F).

**Figure 10:**
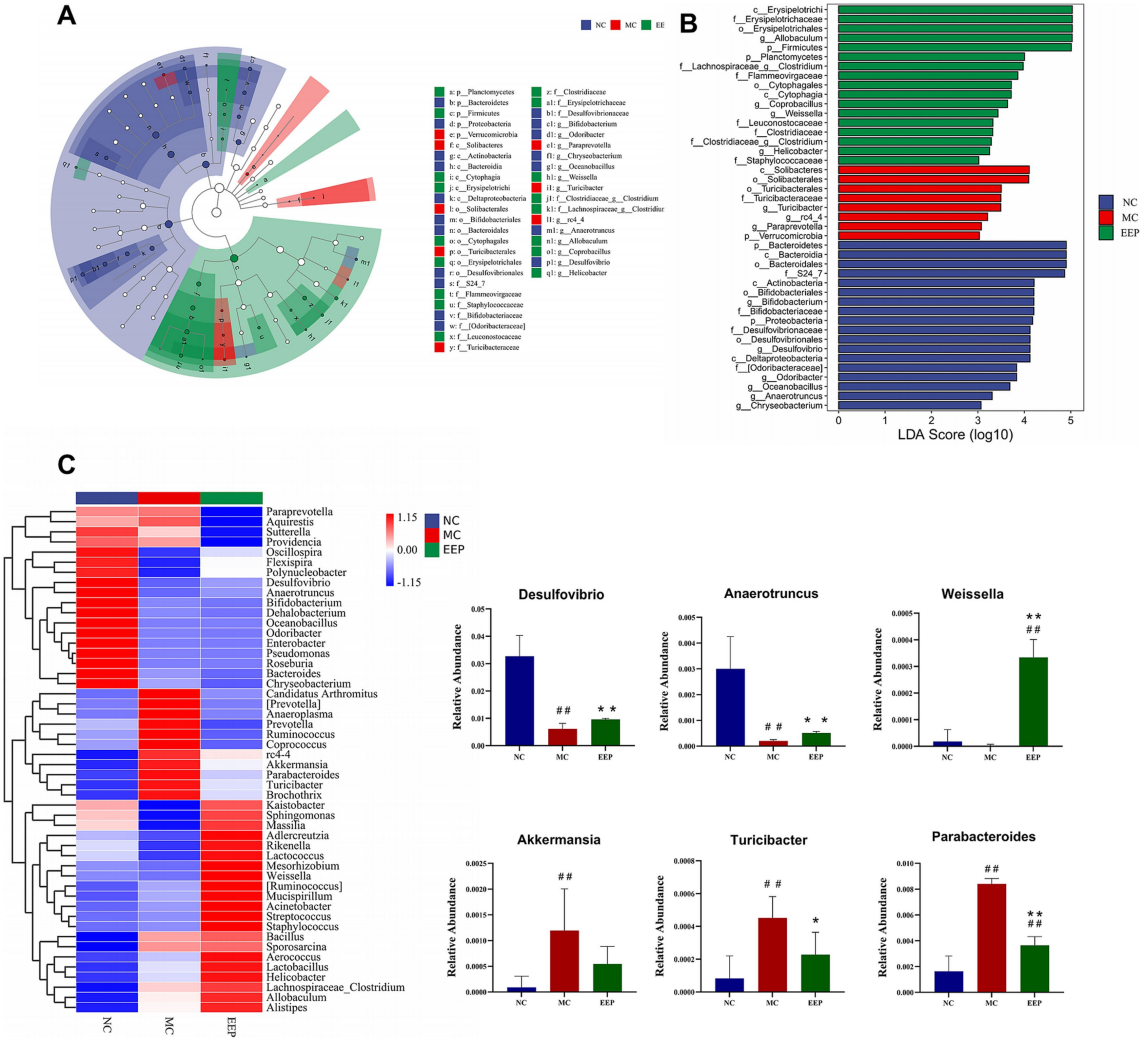
**(A)** LEfSe multi-level species histogram of the mice gut microbiota in each group, **(B)** cladogram. **(C)** Heatmap of top 50 gut microbiota at the genus level and relative abundance of significantly altered bacterial taxa. Data are expressed as mean ± SD (*n* = 6), # *p* < 0.05 and ## *p* < 0.01 compared with NC, * *p* < 0.05 and ** *p* < 0.01 compared with MC.

LEfSe analysis was performed to explore gut microbiota differences among groups from the phylum level to the genus level (LDA score > 3, *p* < 0.05). The cladogram shows that the gut microbiota changed significantly in different taxa (from phylum to genus) after EEP supplementation. As shown in the histograms, *p_Bacteroidetes*, *c_Bacteroidia*, *o_Bacteroidales*, and *f_S24_7* were the representative bacteria in the NC group, *c_Solibacteres*, *o_Solibacterales*, *o_Turicibacterales*, *f_Turicibacteraceae*, and *g_Turicibacter* were the representative bacteria in the MC group, and *c_Erysipelotrichi*, *f_Erysipelotrichaceae*, *o_Erysipelotrichales*, *g_Allobaculum*, and *p_Firmicutes* were the representative bacteria in the EEP group (Figures 10A,B). To further illustrate the differences in microbial abundance between groups, the top 50 most abundant genera were displayed in the heatmap, as shown in Figure 10C, intense exercise significantly altered the relative abundance of certain bacterial genera, such as *Desulfovibrio, Parabacteroides, Anaerotruncus, Akkermansia, Turicibacter,* and *Weissella*. However, the abundance of these genera reversed in the EEP group, suggesting that EEP restored the relative abundance of specific bacterial communities.

**Figure 11:**
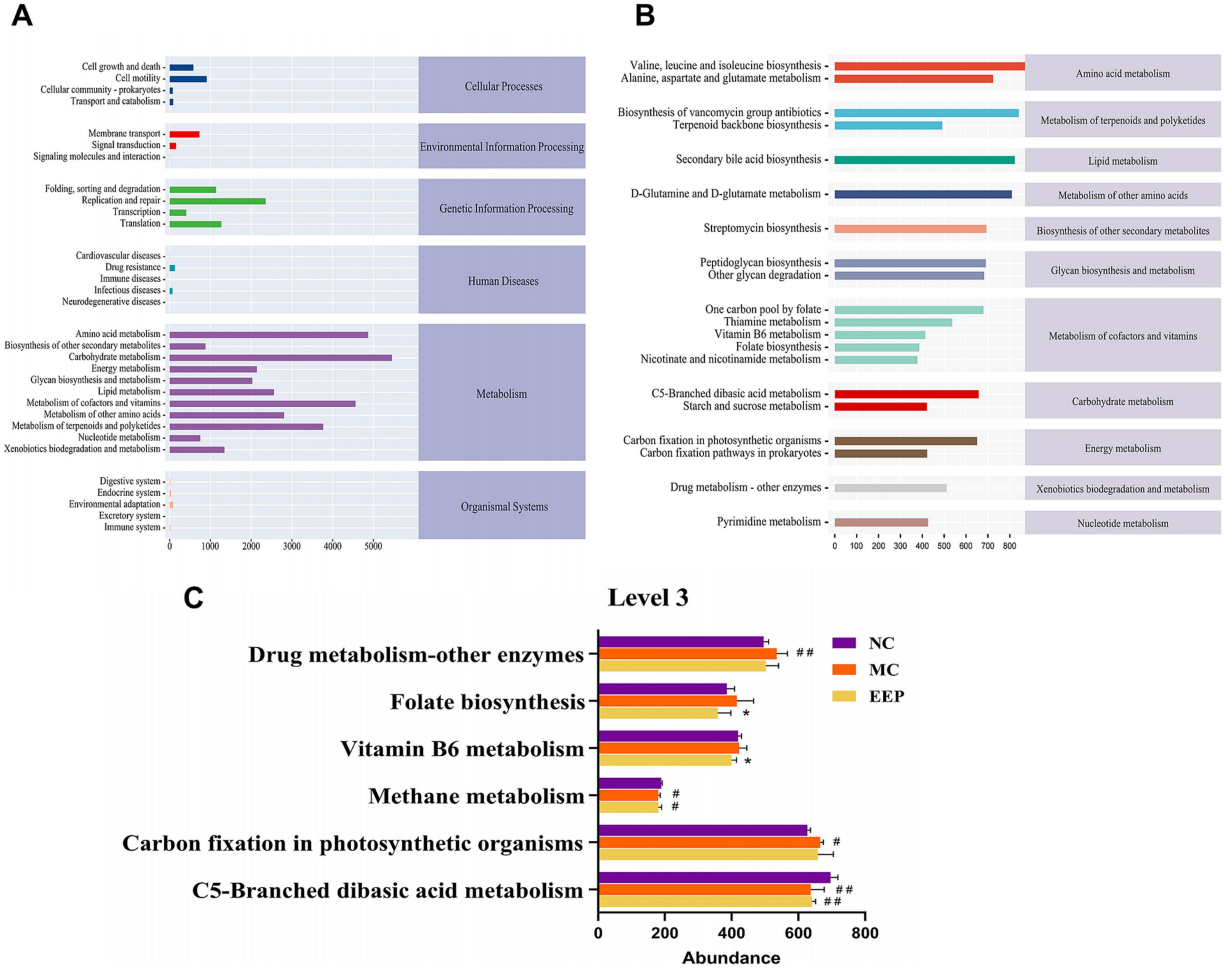
Predicted metabolic functions for the altered genes of gut microbiota. **(A)** Microbial metabolic function of KEGG pathways at level 2, **(B,C)** level 3. Data are expressed as mean ± SD (*n* = 10), # *p* < 0.05 and ## *p* < 0.01 compared with NC, * *p* < 0.05 compared with MC.

To elucidate alterations in microbial functional profiles resulting from changes in the gut microbial community, we conducted a functional analysis of the microbiota using PICRUSt2. At the KEGG pathway classification Level 1, metabolic pathways were the most abundant. At Level 2, the top three most abundant pathways were those involved in metabolizing carbohydrates, amino acids, cofactors, and vitamins (Figure 11A) 0.173 pathways were enriched at Level 3, with the top 20 most abundant pathways shown in Figure 11B. Among these, the abundances of pathways such as drug metabolism, other enzymes, carbon fixation in photosynthetic organisms, folate biosynthesis, vitamin B6 metabolism, methane metabolism, and C5-branched dibasic acid metabolism were considerably altered among the three sample groups (Figure 11C). These findings suggest that EEP intervention may substantially impact gut microbiota function by modulating these metabolic pathways.

### 3.10 Correlation analysis

Orthogonal partial least square (O2PLS) analysis is an advanced method for the bidirectional modeling and predictive analysis of two omics datasets. It allows deep exploration of the intrinsic relationships between these datasets, determines the degree of association between them, and identifies key variables. The loading value is an explanatory power indicator of a variable in each component, where the sign (positive or negative) indicates the association directions, whereas the absolute value reflects the association strength. Additionally, the distance between two points is proportional to their correlation, implying that the closer points exhibit a stronger correlation compared to more distant ones.

**Figure 12:**
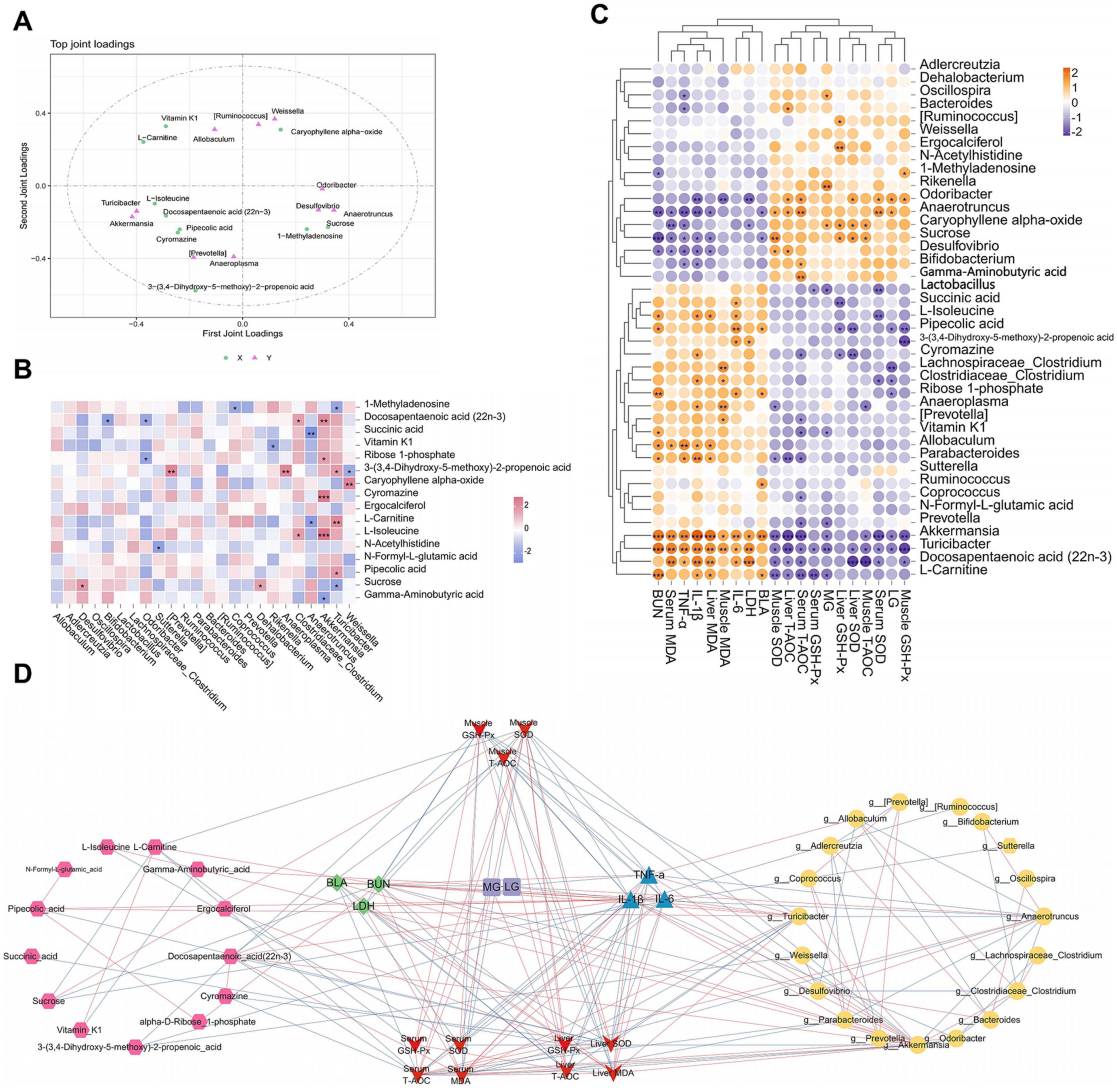
Correlation analysis among specific bacterial genera, differential metabolites, and biochemical indices. **(A)** O2PLS loading diagram of the specific bacterial genera and differential metabolites, **(B)** correlation heatmap. **(C)** Correlation heatmap of biological markers, **(D)** networks diagram.

By calculating the distance of each point from the origin, we identified the top ten variables with the highest weights in each group and generated a loading plot (Figure 12A). This plot demonstrates strong associations between the two omics datasets. Among them, 3-(3,4-dihydroxy-5-methoxy)-2-propenoic acid, L-carnitine, *Akkermansia*, vitamin K1, *Turicibacter*, *Prevotella*, sucrose, *Anaeroplasma*, *Weissella*, and *Anaerotruncus* were the top 10 variables with the highest weights. These variables played critical roles in the association between the two omics datasets. Correlation heatmap analysis between differential metabolites and specific genera confirmed the conclusions from the O2PLS analysis. As shown in Figure 12B, there was a strong correlation between the top 10 variables with the highest weights.

Figures 12C,D reveal the correlations between biochemical indices and different omics results from varying perspectives. In both the correlation heatmap and network diagram, *Akkermansia*, *Turicibacter*, *Anaerotruncus*, sucrose, L-carnitine, pipecolic acid, and L-isoleucine were strongly correlated with biochemical indices. Integrating the results of the three analyses, we concluded that key genera, including *Akkermansia*, *Turicibacter*, and *Anaeroplasma*, as well as differential metabolites such as sucrose, L-carnitine, and pipecolic acid, play pivotal regulatory roles in EEP-mediated alleviation of exercise-induced fatigue in mice.

## 4 Discussion

Exercise-induced fatigue is a complex physiological phenomenon involving multiple mechanisms, including energy metabolism dysregulation, oxidative stress, and inflammatory responses. Although EEP has been shown to have anti-fatigue potential, the precise underlying mechanisms necessitate further investigation. In this study, we used an animal fatigue model coupled with metabolomic and microbiome analyses to explore the impacts and mechanisms of fatigue from a new perspective.

The exhaustive swimming test, known for its high objectivity and repeatability, is widely used in anti-fatigue research (28, 29). In this study, compared to the MC group, the exhaustive swimming time of the EEP group was significantly prolonged by 27.64%, whereas no significant differences were observed in body weight, food intake, or visceral index among the groups. These results indicated that EEP enhanced exercise endurance without compromising the overall mice health (30). Intense exercise significantly reduced LG and MG levels and significantly increased BLA and BUN levels. These findings suggest that rapid depletion of energy reserves activates anaerobic metabolism, leading to lactate accumulation and increased protein catabolism. Moreover, increased LDH levels indicated muscle damage, triggering the release of pro-inflammatory cytokines, including IL-1β, IL-6, and TNF-*α*. These cytokines exacerbate muscle tissue damage and amplify inflammatory responses via oxidative stress pathways (31). Additionally, MDA levels were significantly increased in the skeletal muscles, serum, and liver, concurrently with reduced activities of antioxidant enzymes, such as SOD, GSH-Px, and T-AOC. These results demonstrated that intense exercise induces oxidative stress in multiple tissues. However, EEP supplementation effectively reversed the exercise-induced changes. Collectively, these findings suggest that EEP enhances exercise endurance by alleviating energy depletion, reducing muscle damage, and attenuating both oxidative stress and inflammatory response.

In this study, untargeted metabolomics identified 16 differential metabolites, six of which—GABA, pipecolic acid, L-isoleucine, sucrose, succinic acid, and L-carnitine—exhibited close association with exercise-induced fatigue alleviation. These metabolites significantly influenced seven metabolic pathways, including the TCA cycle, and the metabolism of amino acids and carbohydrates, suggesting that EEP exerts its anti-fatigue effects through multiple pathways. One of the key mechanisms by which EEP alleviates fatigue is through its modulation of the TCA cycle. Intense exercise results in the accumulation of succinic acid due to the ROS-mediated inhibition of mitochondrial complex II (succinate dehydrogenase), thereby disrupting the TCA cycle and reducing ATP production (32–34). The EEP flavonoids and polyphenols enhance antioxidant defenses, reduce ROS levels, and restore succinic acid levels, consequently improving TCA cycle efficiency and mitochondrial function. Moreover, EEP also regulated amino acid and fatty acid metabolism. In the EEP group, L-isoleucine and pipecolic acid levels were reduced, suggesting that EEP may mitigate muscle protein breakdown and optimize energy metabolism (35). EEP restores L-carnitine levels, enhances fatty acid oxidation, and improves energy metabolism (36). Reduced GABA levels in the exercise group reflect diminished glutamate availability, as GABA is synthesized from glutamate via glutamate decarboxylase. EEP supplementation restored this balance, potentially alleviating exercise-induced fatigue by enhancing GABA synthesis from glutamate and stabilizing neurotransmitter levels (37). Furthermore, increased sucrose levels following EEP supplementation indicate enhanced carbohydrate metabolism, reduced reliance on sucrose as an immediate energy source, and improved energy utilization efficiency.

In recent years, the role of gut microbiota in alleviating exercise-induced fatigue has attracted significant attention (37–41). In this study, EEP supplementation significantly altered gut microbiota composition in mice, providing new mechanistic insights into its anti-fatigue effects. At the phylum level, EEP significantly increased the Firmicutes-to-Bacteroidetes ratio, which is consistent with the findings from studies on anti-fatigue compounds rich in flavonoids and polyphenols (37, 41, 42). An increased Firmicutes-to-Bacteroidetes ratio is often associated with enhanced energy metabolism efficiency, suggesting that EEP may improve exercise endurance by modulating energy metabolism. At the genus level, EEP significantly increased the abundance of *Allobaculum* within the Firmicutes phylum to 24.38%, significantly higher than other groups. *Allobaculum* is closely associated with short-chain fatty acid production (SCFAs) (43), which plays crucial roles in maintaining intestinal barrier integrity and regulating systemic inflammation. These findings suggest that EEP may mitigate exercise-induced fatigue by promoting SCFA production, improving gut barrier function, and reducing systemic inflammation. EEP supplementation also led to significant enrichment of other genera, such as *Coprobacillus* and *Clostridium*, observed in patients with chronic fatigue syndrome (44). In contrast, *Sutterella* abundance was significantly lower in the EEP group. *Sutterella* degrades IgA and disrupts the intestinal immune barrier (45). Its reduction may help alleviate intestinal inflammatory stress, indirectly mitigating exercise-induced fatigue. Furthermore, intense exercise significantly altered the relative abundance of certain genera, including *Desulfovibrio*, *Parabacteroides*, *Anaerotruncus*, *Akkermansia*, *Turicibacter*, and *Weissella*. However, EEP supplementation reversed these alterations by restoring the relative abundances of specific bacterial communities. This suggests that EEP counteracts exercise-induced dysbiosis, thereby promoting a healthy gut microbiota profile.

**Figure 13:**
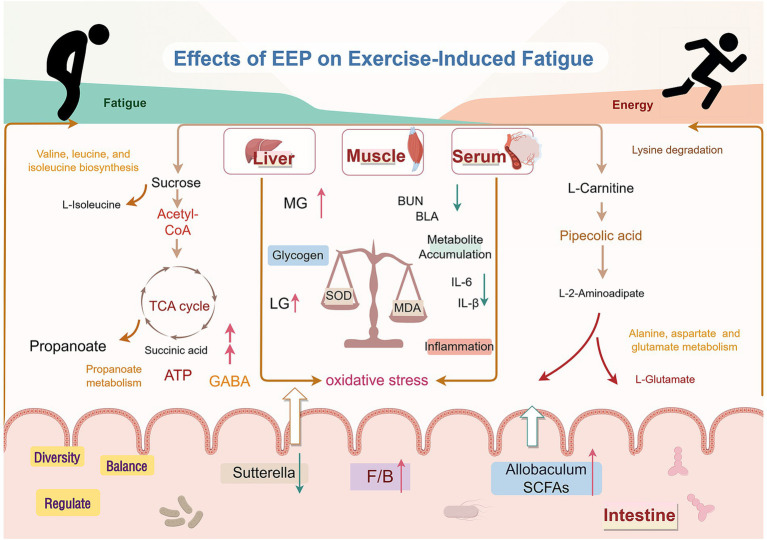
Mechanism of EEP in relieving exercise-induced fatigue. Created with FigDraw.com.

In this study, a comprehensive analysis was conducted to examine the correlations between biochemical indices, differential metabolites, and the gut microbiota, revealing several significant relationships (Figure 13). The correlation analysis demonstrated complex interactions between the metabolites and biochemical indices, further validating the crucial role of these markers in mitigating fatigue. Regarding the gut microbiota, we specifically observed changes in the abundances of *Akkermansia* and *Turicibacter* across different experimental groups. Intense exercise increased the abundance of these bacteria in the MC group, whereas their levels were significantly decreased in the EEP group. This shift aligns with the patterns of oxidative stress, inflammatory responses, and metabolites, such as sucrose, L-carnitine, L-isoleucine, and GABA, suggesting that *Akkermansia* and *Turicibacter* may influence recovery from exercise-induced fatigue by modulating the gut microbiota. *Akkermansia* has been extensively studied for its role in maintaining intestinal barrier integrity, immune regulation, and energy metabolism (46). Our findings further support its potential role in alleviating exercise-induced fatigue. Prior research has indicated that *Turicibacter* is particularly sensitive to oxidative stress, and we hypothesized that it may play an essential role in reducing oxidative muscle damage and systemic inflammation (47).

While this study demonstrated the beneficial impact of EEP on exercise-induced fatigue and elucidated the underlying mechanisms from the perspectives of metabolomics and the gut microbiota, several key questions remain unanswered. These include the specific molecular mechanisms by which EEP alleviates fatigue, the core active constituents responsible for its anti-fatigue effects, and whether these effects are attributed to a single compound or a synergistic combination of components. A thorough investigation of these aspects will enhance our understanding of the anti-fatigue mechanisms of EEP and provide crucial scientific evidence for the effective and safe anti-fatigue product development.

## 5 Conclusion

In this study, we rigorously investigated the multifaceted mechanisms by which EEP alleviates exercise-induced fatigue employing a mouse fatigue model, combined with metabolomic and microbiome analyses. The results demonstrate that EEP significantly prolongs exhaustive swimming time, improves metabolite accumulation, reduces muscle damage, and effectively suppresses oxidative stress and inflammatory responses, which enhances exercise endurance. Metabolomic profiling revealed that EEP regulates key pathways such as the TCA cycle, amino acid, and carbohydrate metabolisms, thereby restoring critical metabolite levels, including GABA, L-isoleucine, and L-carnitine, and providing metabolic support to alleviate fatigue. Microbiome analysis indicated that EEP modulates gut microbiota composition, specifically by increasing the Firmicutes/Bacteroidetes ratio, enhancing the abundance of *Allobaculum*, and reducing *Sutterella* levels, thereby improving gut barrier function and promoting short-chain fatty acid production, which further alleviates systemic inflammation and oxidative stress. In summary, this study reveals the anti-fatigue mechanism of EEP from the perspectives of metabolomics and gut microbiota. However, the molecular mechanisms and active components involved in the anti-fatigue effect of EEP need to be further validated and explored.

## Data Availability

The original contributions presented in the study are included in the article/Supplementary material, further inquiries can be directed to the corresponding author.

## Footnotes

^1^ https://www.genescloud.cn

^2^ http://www.hmdb.ca/metabolites

^3^ https://www.metaboanalyst.ca/

## Glossary

EEP: ethanol extract of propolis
SOD: superoxide dismutase
LG: liver glycogen
PCA: principal component analysis
MG: muscle glycogen
OPLS-DA: orthogonal partial least squares-discriminant analysis
BLA: blood lactate
PCoA: principal coordinates analysis
BUN: blood urea nitrogen
VIP: variables important for projection
LDH: lactate dehydrogenase
FC: fold change
IL-6: interleukin-6
GABA: Gamma-Aminobutyric acid
IL-1β: interleukin-1β
TCA cycle: tricarboxylic acid cycle
TNF-*α*: tumor necrosis factor-alpha
HMDB: Human Metabolome Database
GSH-Px: glutathione peroxidase
KEGG: Kyoto Encyclopedia of Genes and Genomes
T-AOC: total antioxidant capacity
LEfSe: linear discriminate analysis effect size
MDA: malondialdehyde
H&E: hematoxylin and eosin
SCFAs: short-chain fatty acids
O2PLS: orthogonal partial least square
