# Supplementary material for: Ethanol extract of propolis relieves exercise-induced fatigue via modulating the metabolites and gut microbiota in mice
Source: Front Nutr. 2025 Mar 26;12:1549913. doi: 10.3389/fnut.2025.1549913 (PMC11980171; doi:10.3389/fnut.2025.1549913)
Supplement: Supplementary file 1 [file Table_1.DOCX]

Supplementary Material

# Supplementary Figures and Tables

## Supplementary Tables

Table S1 Peak area of top 100 substances

| Name | Formula | Molecular weight (Da) | CAS | RT [min] |
| --- | --- | --- | --- | --- |
| Flavonoids | | | | |
| Acacetin | C16H12O5 | 284.0685 | 480-44-4 | 7.1 |
| Chrysin | C15H10O4 | 254.0579 | 480-40-0 | 6.9 |
| 3-O-Methylquercetin | C16H12O7 | 316.0583 | 1486-70-0 | 5.4 |
| 2',3',4',5,7-Pentahydroxyflavone* | C15H10O7 | 302.0427 | 480-16-0 | 5.2 |
| Tectochrysin | C16H12O4 | 268.0736 | 520-28-5 | 8.5 |
| Artocarpanone | C16H14O6 | 302.079 | 520-25-2 | 5.1 |
| Morin* | C15H10O7 | 302.0427 | 480-16-0 | 4 |
| 3,5,7-Trihydroxyflavanone (Pinobanksin) | C15H12O5 | 272.0685 | 548-82-3 | 5.8 |
| Kaempferide (3,5,7-Trihydroxy-4'-methoxyflavone) | C16H12O6 | 300.0634 | 491-54-3 | 6.3 |
| 5,7,8-Tetrahydroxy-6-methoxyflavone | C16H12O6 | 300.0634 | - | 4.9 |
| 1,8-dihydroxy-2,6-dimethylxanthen-9-one | C15H12O4 | 256.0736 | - | 7 |
| 4',5-Dihydroxy-3,3',7-Trimethoxyflavone; Pachypodol* | C18H16O7 | 344.0896 | 33708-72-4 | 7.6 |
| Ayanin (3',5-Dihydroxy-3,4',7-Trimethoxyflavone)* | C18H16O7 | 344.0896 | 572-32-7 | 7.5 |
| 7,3',4'-Trihydroxyquercetin* | C18H16O7 | 344.0896 | - | 7.4 |
| 6,7,8-Tetrahydroxy-5-methoxyflavone* | C16H12O6 | 300.0634 | - | 6 |
| 2',4'-Dihydroxy-4-methoxychalcone; (E)-Cardamonin | C16H14O4 | 270.0892 | 19309-14-9 | 7.8 |
| Tenaxin I* | C18H16O7 | 344.0896 | 86926-52-5 | 6.8 |
| Pinocembrin (Dihydrochrysin) | C15H12O4 | 256.0736 | 480-39-7 | 7 |
| Eupatilin (5,7-Dihydroxy-3',4',6-Trimethoxyflavone)* | C18H16O7 | 344.0896 | 22368-21-4 | 6.7 |
| Pinostrobin | C16H14O4 | 270.0892 | 480-37-5 | 8.4 |
| 3',7-dihydroxy-4'-methoxyflavone* | C16H12O5 | 284.0685 | 54867-60-6 | 4.9 |
| 3,5,4'-Trihydroxy-7-methoxyflavone (Rhamnocitrin)* | C16H12O6 | 300.0634 | 569-92-6 | 5.8 |
| Galangin (3,5,7-Trihydroxyflavone) | C15H10O5 | 270.0528 | 548-83-4 | 7.1 |
| Diosmetin (5,7,3'-Trihydroxy-4'-methoxyflavone)* | C16H12O6 | 300.0634 | 520-34-3 | 4.3 |
| 5,7,3',5'-tetrahydroxy-6-methylfavanone* | C16H12O6 | 300.0634 | - | 5.7 |
| Prunetin-4'-O-glucoside(Prunitrin)* | C22H22O10 | 446.1213 | 154-36-9 | 4.5 |
| (Z)-4,6-Dihydroxy-2-(4-Hydroxy-3-Methoxybenzylidene)-7-Methylbenzofuran-3(2h)-One* | C17H14O6 | 314.079 | - | 7.2 |
| Tupichinol E* | C17H14O6 | 314.079 | - | 7.3 |
| Chrysin-7-O-glucoside* | C21H20O9 | 416.1107 | 31025-53-3 | 5.1 |
| Chrysin-5-O-glucoside (Toringin)* | C21H20O9 | 416.1107 | 1329-10-8 | 5 |
| Hydroxygenkwanin | C16H12O6 | 300.0634 | 20243-59-8 | 6.5 |
| Wogonin (5,7-Dihydroxy-8-Methoxyflavone) | C16H12O5 | 284.0685 | 632-85-9 | 6.9 |
| Gerontoisoflavone A | C17H14O6 | 314.079 | - | 4.8 |
| 4'-Demethyleucomin glucoside* | C22H22O10 | 446.1213 | - | 4.6 |
| (E)-7-O-β-D-glucopyranoside-5-hydroxy-3-(4'-hydroxybenzylidene)-chroman-4-one* | C22H22O10 | 446.1213 | - | 4.6 |
| Rhamnetin; 3,5,3',4'-Tetrahydroxy-7-Methoxyflavone | C16H12O7 | 316.0583 | 90-19-7 | 6.2 |
| Norartocarpetin | C15H10O6 | 286.0477 | 520-30-9 | 5.6 |
| Quercetin-3,3'-dimethyl ether | C17H14O7 | 330.074 | 4382-17-6 | 6.5 |
| Azaleatin (5-O-Methylquercetin) | C16H12O7 | 316.0583 | 529-51-1 | 4.4 |
| Apigenin; 4',5,7-Trihydroxyflavone | C15H10O5 | 270.0528 | 520-36-5 | 5.7 |
| Loureirin D | C16H16O5 | 288.0998 | 119425-91-1 | 5.1 |
| Nevadensin | C18H16O7 | 344.0896 | 10176-66-6 | 7.1 |
| Drimiopsin C | C15H12O6 | 288.0634 | 773850-90-1 | 4.6 |
| Oroxin A | C21H20O10 | 432.1056 | 57396-78-8 | 4.7 |
| 2-Hydroxy-2,3-dihydrogenistein* | C15H12O6 | 288.0634 | - | 4.9 |
| 3,7-dihydroxy-4'-methoxyflavone | C16H12O5 | 284.0685 | 54867-60-6 | 4.5 |
| Naringenin (5,7,4'-Trihydroxyflavanone) | C15H12O5 | 272.0685 | 480-41-1 | 5.5 |
| Xanthomicrol (5,4'-Dihydroxy-6,7,8-trimethoxyflavone)* | C18H16O7 | 344.0896 | 16545-23-6 | 5 |
| Sophoricoside* | C21H20O10 | 432.1056 | 152-95-4 | 4.4 |
| Phenolic acids | | | | |
| 2-Hydroxycinnamic acid* | C9H8O3 | 164.0473 | 583-17-5 | 4.1 |
| α-Hydroxycinnamic Acid* | C9H8O3 | 164.0473 | 5801-57-0 | 4.2 |
| Phenethyl caffeate | C17H16O4 | 284.1049 | 104594-70-9 | 7.1 |
| Ferulic acid | C10H10O4 | 194.0579 | 537-98-4 | 4 |
| 3,4-Dihydroxybenzoic acid (Protocatechuic acid)* | C7H6O4 | 154.0266 | 99-50-3 | 2.5 |
| 2-(Formylamino)benzoic acid | C8H7NO3 | 165.0426 | 3342-77-6 | 3.8 |
| Caffeic aldehyde | C9H8O3 | 164.0473 | 141632-15-7 | 3.9 |
| Phenyl acetate | C8H8O2 | 136.0524 | 122-79-2 | 4.2 |
| 4-Hydroxybenzoic acid | C7H6O3 | 138.0317 | 99-96-7 | 3 |
| Ethyl caffeate | C11H12O4 | 208.0736 | 102-37-4 | 5.5 |
| Salicylic acid | C7H6O3 | 138.0317 | 69-72-7 | 3.1 |
| p-Coumaric acid | C9H8O3 | 164.0473 | 501-98-4 | 3.8 |
| Eugenol | C10H12O2 | 164.0837 | 97-53-0 | 4 |
| Dibutyl phthalate* | C16H22O4 | 278.1518 | 84-74-2 | 9.6 |
| Phthalic anhydride | C8H4O3 | 148.016 | 85-44-9 | 9.7 |
| Methyl cumalate* | C7H6O4 | 154.0266 | 6018-41-3 | 2.4 |
| 3,4-Dimethoxycinnamic acid | C11H12O4 | 208.0736 | 2316-26-9 | 4.9 |
| 2,5-Dihydroxybenzoic acid; Gentisic Acid* | C7H6O4 | 154.0266 | 490-79-9 | 2.6 |
| 1,3-O-Di-p-Coumaroylglycerol | C21H20O7 | 384.1209 | - | 5.8 |
| Butyl isobutyl phthalate* | C16H22O4 | 278.1518 | 17851-53-5 | 9.6 |
| Lipids | | | | |
| γ-Linolenic Acid* | C18H30O2 | 278.2246 | 506-26-3 | 10.8 |
| α-Linolenic Acid* | C18H30O2 | 278.2246 | 463-40-1 | 10.7 |
| Lyngbic acid | C15H28O3 | 256.2038 | 70607-97-5 | 7.3 |
| 2R-hydroxy-9Z,12Z,15Z-octadecatrienoic acid | C18H30O3 | 294.2195 | - | 9.4 |
| 3-Hydroxyoctadecanoic Acid | C18H36O3 | 300.2664 | 45261-96-9 | 10.2 |
| Choline Alfoscerate | C8H20NO6P | 257.1028 | 28319-77-9 | 0.8 |
| 9,12-Octadecadiynoic Acid | C18H28O2 | 276.2089 | 2012-14-8 | 9.2 |
| Stearic Acid | C18H36O2 | 284.2715 | 1957/11/4 | 10.8 |
| Octadeca-11E,13E,15Z-trienoic acid | C18H30O2 | 278.2246 | 25575-00-2 | 9.6 |
| 12(13)Ep-9-KODE | C18H30O4 | 310.2144 | 478931-82-7 | 7.7 |
| Others | | | | |
| Machilusolide D | C18H30O3 | 294.2195 | - | 9.3 |
| 6-Hydroxyrhein | C15H8O7 | 300.027 | - | 6 |
| 1,5-Dihydroxy-2,3-Dimethoxy-10-Methyl-9-Acridone | C16H15NO5 | 301.095 | - | 6.2 |
| Tri-p-coumaroyl Spermidine | C34H37N3O6 | 583.2677 | - | 5.2 |
| Erythro-Magnolignan B | C18H20O5 | 316.1311 | - | 5.4 |
| Betaine | C5H11NO2 | 117.079 | 107-43-7 | 0.8 |
| 4-Methylbenzaldehyde* | C8H8O | 120.0575 | 104-87-0 | 4.2 |
| Chrysophanol-1-O-β-D-glucoside* | C21H20O9 | 416.1107 | 4839-60-5 | 5.2 |
| 3-Methylbenzaldehyde* | C8H8O | 120.0575 | 620-23-5 | 4.3 |
| 4-Hydroxyacetophenone | C8H8O2 | 136.0524 | 99-93-4 | 4 |
| 4-Guanidinobutyric acid | C5H11N3O2 | 145.0851 | 463-00-3 | 0.8 |
| 4-hydroxyphenyl acrylaldehyde | C9H8O3 | 164.0473 | - | 4 |
| 2,5-Dihydroxybenzaldehyde* | C7H6O3 | 138.0317 | 1194-98-5 | 3.3 |
| Azelaic acid | C9H16O4 | 188.1049 | 123-99-9 | 4.4 |
| Protocatechualdehyde* | C7H6O3 | 138.0317 | 139-85-5 | 3.3 |
| 6-Demethoxy-7-methylcapillarisin | C16H12O6 | 300.0634 | - | 7 |
| 3,8-Dihydroxy-1-methoxy-9,10-anthraquinone glucoside | C21H20O10 | 432.1051 | - | 4.9 |
| 4-Hydroxybenzaldehyde | C7H6O2 | 122.0368 | 123-08-0 | 3.7 |
| L-Lysine-Butanoic Acid | C10H22N2O4 | 234.158 | 80407-71-2 | 0.8 |
| D-Fructose 6-phosphate* | C6H13O9P | 260.0297 | 643-13-0 | 1 |
| 3α,12β,15α,21β,24-pentahydroxyserratane | C30H50O8 | 538.3506 | - | 11.2 |

Table S2 Differential metabolite information between NC group and MC group

| Compound Name | VIP | Fold Change | M/Z | Trends | Formula | Precursor Type |
| --- | --- | --- | --- | --- | --- | --- |
| N-Acetyl-L-glutamine | 1.0265 | 1.5031 | 187.0709 | Up | C_7_H_12_N_2_O_4_ | [M-H]- |
| Nonadecanoic acid | 1.0895 | 1.3596 | 297.2775 | Up | C_19_H_38_O_2_ | [M-H]- |
| all-trans-Retinoic acid | 1.0909 | 1.7407 | 299.1996 | Up | C_20_H_28_O_2_ | [M-H]- |
| N-Formyl-L-glutamic acid | 1.0920 | 1.3083 | 176.0716 | Up | C_6_H_9_NO_5_ | [M+H]+ |
| Leucine | 1.1161 | 1.2942 | 130.0860 | Up | C_6_H_13_NO_2_ | [M-H]- |
| Daidzin | 1.1263 | 1.4459 | 397.2222 | Up | C_21_H_20_O_9_ | [M-H_2_O-H]- |
| Epinephrine | 1.1356 | 1.5181 | 184.0952 | Up | C_9_H_13_NO_3_ | [M+H]+ |
| D-Lyxose | 1.1470 | 1.9378 | 149.9935 | Up | C_5_H_10_O_5_ | [M]- |
| Linoleic acid | 1.1472 | 1.3652 | 280.2646 | Up | C_18_H_32_O_2_ | [M]+ |
| Chenodeoxycholic acid | 1.1537 | 1.5244 | 391.2826 | Up | C_24_H_40_O_4_ | [M-H]- |
| Oxalacetic acid | 1.1563 | 1.2943 | 112.9843 | Up | C_4_H_4_O_5_ | [M-H_2_O-H]- |
| 11Z-Eicosenoic acid | 1.1833 | 1.3554 | 310.2815 | Up | C_20_H_38_O_2_ | [M]- |
| N-Succinyl-L-citrulline | 1.2057 | 2.0296 | 276.1195 | Up | C_10_H_17_N_3_O_6_ | [M+H]+ |
| Vitamin K1 | 1.2126 | 2.6155 | 449.0934 | Up | C_31_H_46_O_2_ | [M-H]- |
| 8-Amino-7-oxononanoate | 1.2694 | 1.9029 | 188.1290 | Up | C_9_H_17_NO_3_ | [M+H]+ |
| Aminoadipic acid | 1.2783 | 1.5051 | 160.0606 | Up | C_6_H_11_NO_4_ | [M-H]- |
| Betaine | 1.2800 | 1.2157 | 118.0868 | Up | C_5_H_11_NO_2_ | [M+H]+ |
| (-)-Isopiperitenone | 1.2814 | 1.4502 | 150.1030 | Up | C_10_H_14_O | [M]+ |
| alpha-D-Ribose 1-phosphate | 1.3013 | 1.5744 | 229.0098 | Up | C_5_H_11_O_8_P | [M-H]- |
| 1-palmitoylglycerophosphocholine | 1.3026 | 1.5148 | 496.3419 | Up | C_24_H_51_NO_7_P | [M]+ |
| Glutathione | 1.3106 | 1.4247 | 307.0840 | Up | C_10_H_17_N_3_O_6_S | [M]+ |
| L-Lysine | 1.3194 | 1.2109 | 147.1128 | Up | C_6_H_14_N_2_O_2_ | [M+H]+ |
| Cyromazine | 1.3203 | 1.2239 | 166.0982 | Up | C_6_H_10_N_6_ | [M]+ |
| Sphingosine | 1.3411 | 1.2322 | 300.2905 | Up | C_18_H_37_NO_2_ | [M+H]+ |
| 17a-Estradiol | 1.3437 | 1.5440 | 272.1861 | Up | C_18_H_24_O_2_ | [M]+ |
| 3-Oxo-5beta-cholanate | 1.3696 | 2.4526 | 374.2745 | Up | C_24_H_38_O_3_ | [M]+ |
| beta-D-Fructose 6-phosphate | 1.3768 | 1.7495 | 259.0195 | Up | C_6_H_13_O_9_P | [M-H]- |
| 5,7-Dihydroxyflavone | 1.3829 | 1.8236 | 253.0492 | Up | C_15_H_10_O_4_ | [M-H]- |
| Pyroglutamic acid | 1.4002 | 1.4561 | 128.0350 | Up | C_5_H_7_NO_3_ | [M-H]- |
| Salicyluric acid | 1.4004 | 1.4589 | 194.0445 | Up | C_9_H_9_NO_4_ | [M-H]- |
| Succinic acid | 1.4017 | 1.6036 | 117.0549 | Up | C_4_H_6_O_4_ | [M-H]- |
| Thymidine | 1.4148 | 1.6883 | 223.0270 | Up | C_10_H_14_N_2_O_5_ | [M-H_2_O-H]- |
| Anserine | 1.4158 | 1.4991 | 240.1813 | Up | C_10_H_16_N_4_O_3_ | [M]+ |
| threo-3-Hydroxy-D-aspartate | 1.4279 | 1.2368 | 149.0233 | Up | C_4_H_7_NO_5_ | [M]+ |
| 8-HETE | 1.4416 | 1.4252 | 303.2325 | Up | C_20_H_32_O_3_ | [M+H-H_2_O]+ |
| Maltol | 1.4453 | 1.9528 | 127.0387 | Up | C_6_H_6_O_3_ | [M+H]+ |
| Picolinic acid | 1.4519 | 1.2227 | 123.0799 | Up | C_6_H_5_NO_2_ | [M]+ |
| Eicosapentaenoic Acid | 1.4532 | 1.4619 | 302.2157 | Up | C_20_H_30_O_2_ | [M]+ |
| 2-Deoxystreptamine | 1.4672 | 1.8398 | 163.1118 | Up | C_6_H_14_N_2_O_3_ | [M+H]+ |
| 4-Pyridoxic acid | 1.4712 | 3.2022 | 182.0438 | Up | C_8_H_9_NO_4_ | [M-H]- |
| 3-(3,4-Dihydroxy-5-methoxy)-2-propenoic acid | 1.4714 | 1.9878 | 193.0493 | Up | C_10_H_10_O_5_ | [M+H-H_2_O]+ |
| Citrulline | 1.4771 | 1.3425 | 174.0872 | Up | C_6_H_13_N_3_O_3_ | [M-H]- |
| Tridecanoic acid | 1.4879 | 2.3664 | 213.1846 | Up | C_13_H_26_O_2_ | [M-H]- |
| Indolepyruvate | 1.5269 | 1.2259 | 204.0664 | Up | C_11_H_9_NO_3_ | [M+H]+ |
| L-Isoleucine | 1.5343 | 1.6272 | 132.0533 | Up | C_6_H_13_NO_2_ | [M+H]+ |
| Coniferyl aldehyde | 1.5559 | 1.6004 | 178.0594 | Up | C_10_H_10_O_3_ | [M]+ |
| L-Carnitine | 1.5619 | 1.8189 | 162.0565 | Up | C_7_H_15_NO_3_ | [M+H]+ |
| Indoleacetaldehyde | 1.5974 | 1.6081 | 160.0767 | Up | C_10_H_9_NO | [M+H]+ |
| Vaccenic acid | 1.6087 | 1.3139 | 281.2434 | Up | C_18_H_34_O_2_ | [M-H]- |
| 13(S)-HpOTrE | 1.6375 | 2.6113 | 311.2225 | Up | C_18_H_30_O_4_ | [M+H]+ |
| 16-Hydroxy hexadecanoic acid | 1.6411 | 2.0129 | 271.2259 | Up | C_16_H_32_O_3_ | [M-H]- |
| Dodecanedioic acid | 1.6579 | 4.2965 | 229.1430 | Up | C_12_H_22_O_4_ | [M-H]- |
| Stearolic acid | 1.6795 | 2.8093 | 263.2380 | Up | C_18_H_32_O_2_ | [M+H-H_2_O]+ |
| Pipecolic acid | 1.6797 | 1.5311 | 130.0502 | Up | C_6_H_11_NO_2_ | [M+H]+ |
| Docosapentaenoic acid (22n-3) | 1.6868 | 1.7935 | 329.2476 | Up | C_22_H_34_O_2_ | [M-H]- |
| 5-Hydroxyindoleacetic acid | 1.7005 | 1.4096 | 191.1064 | Up | C_10_H_9_NO_3_ | [M]- |
| Pyridoxal phosphate | 1.7203 | 1.3265 | 247.1685 | Up | C_8_H_10_NO_6_P | [M]- |
| Hippuric acid | 1.7438 | 1.4943 | 180.0670 | Up | C_9_H_9_NO_3_ | [M+H]+ |
| Pantothenol | 1.7815 | 1.4419 | 206.1396 | Up | C_9_H_19_NO_4_ | [M+H]+ |
| N-Acetyl-L-phenylalanine | 1.7890 | 1.9902 | 206.0806 | Up | C_11_H_13_NO_3_ | [M-H]- |
| S-Hexyl-glutathione | 1.8306 | 2.4558 | 391.2856 | Up | C_16_H_29_N_3_O_6_S | [M]+ |
| Aflatoxin B1 | 1.8920 | 1.5227 | 312.3632 | Up | C_17_H_12_O_6_ | [M]+ |
| Gluconic acid | 1.9915 | 3.7450 | 195.0493 | Up | C_6_H_12_O_7_ | [M-H]- |
| Ketoleucine | 2.0035 | 1.9690 | 131.0692 | Up | C_6_H_10_O_3_ | [M+H]+ |
| Methyleugenol | 2.0266 | 2.2088 | 179.1075 | Up | C_11_H_14_O_2_ | [M+H]+ |
| L-Leucine | 2.0417 | 1.8691 | 130.0861 | Up | C_6_H_13_NO_2_ | [M-H]- |
| Phenylethylamine | 2.0796 | 4.6136 | 121.0654 | Up | C_8_H_11_N | [M]- |
| S-Adenosylmethionine | 2.0960 | 2.5572 | 398.2404 | Up | C_15_H_22_N_6_O_5_S | [M]+ |
| Pyridoxamine | 2.1743 | 1.9452 | 169.0952 | Up | C_8_H_12_N_2_O_2_ | [M+H]+ |
| Heptanoic acid | 2.2482 | 2.1420 | 130.0654 | Up | C_7_H_14_O_2_ | [M]+ |
| Maleamate | 2.2636 | 2.4353 | 115.0030 | Up | C_4_H_5_NO_3_ | [M]- |
| 3-Methylxanthine | 1.0320 | 0.5221 | 149.0456 | Down | C_6_H_6_N_4_O_2_ | [M+H-H_2_O]+ |
| Mibefradil | 1.0847 | 0.7354 | 496.3038 | Down | C_29_H_38_FN_3_O_3_ | [M+H]+ |
| 2-Aminobenzoic acid | 1.0847 | 0.7492 | 137.0462 | Down | C_7_H_7_NO_2_ | [M]+ |
| D-beta-Phenylalanine | 1.0895 | 0.8204 | 166.0874 | Down | C_9_H_11_NO_2_ | [M+H]+ |
| L-Fucose | 1.0976 | 0.6340 | 165.1140 | Down | C_6_H_12_O_5_ | [M+H]+ |
| Azelaic acid | 1.1019 | 0.6483 | 171.0926 | Down | C_9_H_16_O_4_ | [M+H-H_2_O]+ |
| N-Acetylhistidine | 1.1028 | 0.6198 | 198.0856 | Down | C_8_H_11_N_3_O_3_ | [M+H]+ |
| Docosatetraenoyl Ethanolamide | 1.1217 | 0.6560 | 376.3195 | Down | C_24_H_41_NO_2_ | [M+H]+ |
| Biochanin A | 1.1319 | 0.7171 | 283.1678 | Down | C_16_H_12_O_5_ | [M-H]- |
| Iminoarginine | 1.1453 | 0.7389 | 172.0964 | Down | C_6_H_12_N_4_O_2_ | [M]- |
| Dihydrouracil | 1.1788 | 0.7124 | 115.0510 | Down | C_4_H_6_N_2_O_2_ | [M+H]+ |
| Adenosine | 1.1960 | 0.7392 | 268.1033 | Down | C_10_H_13_N_5_O_4_ | [M+H]+ |
| D-Ribose | 1.2088 | 0.8125 | 151.0363 | Down | C_5_H_10_O_5_ | [M+H]+ |
| D-synephrine | 1.2100 | 0.6903 | 168.0918 | Down | C_9_H_13_NO_2_ | [M+H]+ |
| Gamma-Tocotrienol | 1.2132 | 0.6782 | 410.3186 | Down | C_28_H_42_O_2_ | [M]+ |
| Cortisol | 1.2282 | 0.7446 | 361.1993 | Down | C_21_H_30_O_5_ | [M-H]- |
| Ergocalciferol | 1.2432 | 0.6833 | 396.3444 | Down | C_28_H_44_O | [M]+ |
| N-Acetylleucine | 1.2464 | 0.7342 | 174.1134 | Down | C_8_H_15_NO_3_ | [M+H]+ |
| 1-Methyladenosine | 1.2503 | 0.7645 | 281.1204 | Down | C_11_H_15_N_5_O_4_ | [M]- |
| Cortexolone | 1.2613 | 0.7285 | 346.3311 | Down | C_21_H_30_O_4_ | [M]+ |
| 3,4-Methylenedioxyamphetamine | 1.2696 | 0.4491 | 194.1188 | Down | C_11_H_15_NO_2_ | [M+H]+ |
| Taurine | 1.2898 | 0.8316 | 124.0066 | Down | C_2_H_7_NO_3_S | [M-H]- |
| (R)-Pantolactone | 1.3021 | 0.8088 | 131.5340 | Down | C_6_H_10_O_3_ | [M+H]+ |
| gamma-Aminobutyric acid | 1.3505 | 0.6805 | 103.0551 | Down | C_4_H_9_NO_2_ | [M]+ |
| D-Xylose | 1.3895 | 0.6839 | 151.0621 | Down | C_5_H_10_O_5_ | [M+H]+ |
| Retinol | 1.4425 | 0.6415 | 269.2271 | Down | C_20_H_30_O | [M+H-H_2_O]+ |
| Caryophyllene alpha-oxide | 1.4603 | 0.7965 | 221.1909 | Down | C_15_H_24_O | [M+H]+ |
| Pyrimidodiazepine | 1.4925 | 0.7725 | 221.0931 | Down | C_9_H_11_N_5_O_2_ | [M]+ |
| L-Proline | 1.4969 | 0.7291 | 116.0718 | Down | C_5_H_9_NO_2_ | [M+H]+ |
| O-Acetylcarnitine | 1.5040 | 0.5768 | 205.1262 | Down | C_9_H_18_NO_4_ | [M+H]+ |
| Glyceric acid | 1.5191 | 0.4462 | 105.0186 | Down | C_3_H_6_O_4_ | [M-H]- |
| Phosphonoacetate | 1.5605 | 0.6344 | 140.0686 | Down | C_2_H_5_O_5_P | [M]+ |
| Chavicol | 1.6380 | 0.7135 | 135.0808 | Down | C_9_H_10_O | [M+H]+ |
| Cholesterol | 1.7058 | 0.6977 | 369.3524 | Down | C_27_H_46_O | [M+H-H_2_O]+ |
| N-Acetylglutamic acid | 1.7759 | 0.3948 | 190.0505 | Down | C_7_H_11_NO_5_ | [M+H]+ |
| 7,8-Diaminononanoate | 1.8504 | 0.8296 | 189.1647 | Down | C_9_H_20_N_2_O_2_ | [M+H]+ |
| L-Kynurenine | 1.8598 | 0.6290 | 209.0930 | Down | C_10_H_12_N_2_O_3_ | [M+H]+ |
| Sucrose | 1.9759 | 0.7405 | 343.2981 | Down | C_12_H_22_O_11_ | [M+H]+ |
| Antibiotic JI-20A | 1.9873 | 0.7142 | 482.3239 | Down | C_19_H_39_N_5_O_9_ | [M+H]+ |
| Ureidopropionic acid | 2.0573 | 0.6045 | 133.0614 | Down | C_4_H_8_N_2_O_3_ | [M+H]+ |

Table S3 Differential metabolite information between MC group and EEP group

| Compound Name | VIP | Fold Change | M/Z | Trends | Formula | Precursor Type |
| --- | --- | --- | --- | --- | --- | --- |
| m-Cresol | 1.2513 | 1.5897 | 109.1016 | Up | C_7_H_8_O | [M+H]+ |
| 1-Deoxy-D-xylulose | 1.6209 | 1.3998 | 117.0574 | Up | C_5_H_10_O_4_ | [M+H-H_2_O]+ |
| trans-Cinnamate | 1.4117 | 1.4441 | 131.0499 | Up | C_9_H_8_O_2_ | [M+H-H_2_O]+ |
| Anabasine | 1.7499 | 1.3469 | 144.9824 | Up | C_10_H_14_N_2_ | [M+H-H_2_O]+ |
| 4-Hydroxycinnamic acid | 1.1203 | 1.3127 | 146.9810 | Up | C_9_H_8_O_3_ | [M+H-H_2_O]+ |
| Allantoin | 1.2473 | 1.2190 | 158.0462 | Up | C_4_H_6_N_4_O_3_ | [M]+ |
| Aminoadipic acid | 1.1945 | 1.3550 | 160.0606 | Up | C_6_H_11_NO_4_ | [M-H]- |
| 3-(2-Hydroxyphenyl)propanoic acid | 2.0156 | 2.3171 | 167.0708 | Up | C_9_H_10_O_3_ | [M+H]+ |
| DL-Glycerol 1-phosphate | 1.6803 | 2.6921 | 171.0052 | Up | C_3_H_9_O_6_P | [M-H]- |
| D-Galactose | 1.8494 | 2.3476 | 181.0151 | Up | C_6_H_12_O_6_ | [M+H]+ |
| N-Succinyl-L-citrulline | 1.6085 | 1.7759 | 276.1195 | Up | C_10_H_17_N_3_O_6_ | [M+H]+ |
| Citric acid | 1.3674 | 1.3166 | 191.0177 | Up | C_6_H_8_O_7_ | [M-H]- |
| D-Glucuronic Acid | 1.1263 | 2.9108 | 195.1233 | Up | C_6_H_10_O_7_ | [M+H]+ |
| Indolelactic acid | 1.4903 | 1.9784 | 206.0824 | Up | C_11_H_11_NO_3_ | [M+H]+ |
| Capsidiol | 1.1840 | 2.5100 | 219.1756 | Up | C_15_H_24_O_2_ | [M+H-H_2_O]+ |
| Uridine | 1.1530 | 2.0054 | 243.0605 | Up | C_9_H_12_N_2_O_6_ | [M-H]- |
| beta-Alanyl-L-arginine | 1.3025 | 1.4170 | 245.1506 | Up | C_9_H_19_N_5_O_3_ | [M]+ |
| Alprenolol | 1.7472 | 2.7887 | 250.1787 | Up | C_15_H_23_NO_2_ | [M+H]+ |
| Docosatetraenoyl Ethanolamide | 1.0372 | 1.2061 | 376.3195 | Up | C_24_H_41_NO_2_ | [M+H]+ |
| Inosine | 1.0618 | 1.3092 | 267.0735 | Up | C_10_H_12_N_4_O_5_ | [M-H]- |
| gamma-Aminobutyric acid | 1.0873 | 1.2878 | 103.0551 | Up | C_4_H_9_NO_2_ | [M]+ |
| (R)-Pantolactone | 1.1080 | 1.2766 | 131.5340 | Up | C_6_H_10_O_3_ | [M+H]+ |
| Catechin | 1.5583 | 1.6163 | 289.0700 | Up | C_15_H_14_O_6_ | [M-H]- |
| Epiandrosterone | 1.0656 | 1.2914 | 288.9504 | Up | C_19_H_30_O_2_ | [M-H]- |
| 1-Methyladenosine | 1.3195 | 1.3458 | 281.1204 | Up | C_11_H_15_N_5_O_4_ | [M]- |
| Antibiotic JI-20A | 1.2728 | 1.2449 | 482.3239 | Up | C_19_H_39_N_5_O_9_ | [M+H]+ |
| Ergocalciferol | 1.3155 | 1.6074 | 396.3444 | Up | C_28_H_44_O | [M]+ |
| Corticosterone | 1.9976 | 3.0597 | 347.2205 | Up | C_21_H_30_O_4_ | [M+H]+ |
| (4Z,7Z,10Z,13Z,16Z,19Z)-Docosahexaenoic acid ethyl ester | 1.5894 | 2.4738 | 357.2785 | Up | C_24_H_36_O_2_ | [M+H]+ |
| Nitrendipine | 1.8242 | 1.9334 | 361.1401 | Up | C_18_H_20_N_2_O_6_ | [M+H]+ |
| 11-Dehydro-thromboxane B2 | 1.5942 | 2.1704 | 369.2254 | Up | C_20_H_32_O_6_ | [M+H]+ |
| Caryophyllene alpha-oxide | 1.6995 | 1.3725 | 221.1909 | Up | C_15_H_24_O | [M+H]+ |
| LysoPA(16_0_0_0) | 1.1889 | 1.4152 | 409.2322 | Up | C_19_H_39_O_7_P | [M-H]- |
| Sucrose | 1.9588 | 1.3122 | 343.2981 | Up | C_12_H_22_O_11_ | [M+H]+ |
| N-Acetylhistidine | 2.1185 | 2.5661 | 198.0856 | Up | C_8_H_11_N_3_O_3_ | [M+H]+ |
| alpha-D-Ribose 1-phosphate | 1.0622 | 0.7881 | 229.0098 | Down | C_5_H_11_O_8_P | [M-H]- |
| S-Adenosylmethionine | 1.0085 | 0.7781 | 398.2404 | Down | C_15_H_22_N_6_O_5_S | [M]+ |
| Coniferyl aldehyde | 1.1302 | 0.7315 | 178.0594 | Down | C_10_H_10_O_3_ | [M]+ |
| L-Isoleucine | 1.2030 | 0.7382 | 132.0533 | Down | C_6_H_13_NO_2_ | [M+H]+ |
| Vitamin K1 | 1.2001 | 0.4476 | 449.0934 | Down | C_31_H_46_O_2_ | [M-H]- |
| 4-Hydroxyphenylacetaldehyde | 1.1392 | 0.7007 | 136.0488 | Down | C_8_H_8_O_2_ | [M]+ |
| Tyramine | 1.4884 | 0.3622 | 137.0828 | Down | C_8_H_11_NO | [M]+ |
| Spermidine | 1.1068 | 0.7770 | 143.9148 | Down | C_7_H_19_N_3_ | [M-H]- |
| Cyromazine | 1.1977 | 0.8258 | 166.0982 | Down | C_6_H_10_N_6_ | [M]+ |
| 3-Hydroxyanthranilic acid | 1.8255 | 0.5461 | 152.0344 | Down | C_7_H_7_NO_3_ | [M-H]- |
| 2-Amino-4-nitrotoluene | 1.3152 | 0.5906 | 153.0664 | Down | C_7_H_8_N_2_O_2_ | [M+H]+ |
| D-Xylitol | 1.5483 | 0.6832 | 153.0773 | Down | C_5_H_12_O_5_ | [M+H]+ |
| 2,3-Butanediol | 1.3600 | 0.6677 | 155.1079 | Down | C_4_H_10_O_2_S_2_ | [M+H]+ |
| Pipecolic acid | 1.3665 | 0.7325 | 130.0502 | Down | C_6_H_11_NO_2_ | [M+H]+ |
| 3,4-Dihydroxyphenylglycol | 1.8763 | 0.4832 | 171.0643 | Down | C_8_H_10_O_4_ | [M+H]+ |
| 3-Dehydroshikimate | 2.1835 | 0.4689 | 171.9922 | Down | C_7_H_8_O_5_ | [M]+ |
| Tetrahydrodipicolinate | 1.2960 | 0.7855 | 172.0763 | Down | C_7_H_9_NO_4_ | [M+H]+ |
| N-Formyl-L-glutamic acid | 1.5808 | 0.5686 | 176.0716 | Down | C_6_H_9_NO_5_ | [M+H]+ |
| D-Fructose | 1.0068 | 0.7760 | 181.0868 | Down | C_6_H_12_O_6_ | [M+H]+ |
| myo-Inositol | 1.1704 | 0.4827 | 181.0149 | Down | C_6_H_12_O_6_ | [M+H]+ |
| Undecanoic acid | 1.6958 | 0.7476 | 186.9568 | Down | C_11_H_22_O_2_ | [M+H]+ |
| 3-Indoleacrylate | 1.6326 | 0.4416 | 188.0714 | Down | C_11_H_9_NO_2_ | [M+H]+ |
| Methyl (indol-3-yl)acetate | 1.8146 | 0.6479 | 189.0791 | Down | C_11_H_11_NO_2_ | [M]+ |
| Kynurenic acid | 1.3136 | 0.6862 | 190.0868 | Down | C_10_H_7_NO_3_ | [M+H]+ |
| Quinate | 1.4097 | 0.6464 | 191.0550 | Down | C_7_H_12_O_6_ | [M-H]- |
| Diaminopimelic acid | 1.5357 | 0.5530 | 191.0404 | Down | C_7_H_14_N_2_O_4_ | [M+H]+ |
| Scopoletin | 1.0714 | 0.4148 | 193.0493 | Down | C_10_H_8_O_4_ | [M+H]+ |
| 3-Methyl-L-tyrosine | 1.4378 | 0.8262 | 195.1024 | Down | C_10_H_13_NO_3_ | [M]+ |
| Procollagen 5-hydroxy-L-lysine | 1.8283 | 0.2637 | 197.8068 | Down | C_7_H_13_N_3_O_3_R_2_ | [M-H]- |
| Docosapentaenoic acid (22n-3) | 1.5777 | 0.7806 | 329.2476 | Down | C_22_H_34_O_2_ | [M-H]- |
| 3-(3,4-Dihydroxy-5-methoxy)-2-propenoic acid | 1.7788 | 0.4057 | 193.0493 | Down | C_10_H_10_O_5_ | [M+H-H_2_O]+ |
| L-Carnitine | 1.7964 | 0.7434 | 162.0565 | Down | C_7_H_15_NO_3_ | [M+H]+ |
| Myristoleic acid | 1.1270 | 0.8121 | 209.1908 | Down | C_14_H_26_O_2_ | [M+H-H_2_O]+ |
| Succinic acid | 2.0029 | 0.8183 | 117.0549 | Down | C_4_H_6_O_4_ | [M-H]- |
| N-Acetyl-D-glucosamine | 1.5346 | 0.8183 | 221.1532 | Down | C_8_H_15_NO_6_ | [M]- |
| Deoxyuridine | 1.4537 | 0.8015 | 228.1962 | Down | C_9_H_12_N_2_O_5_ | [M]+ |
| 13(S)-HpOTrE | 1.9691 | 0.2328 | 311.2225 | Down | C_18_H_30_O_4_ | [M+H]+ |
| Palmitic acid | 1.4579 | 0.6201 | 256.2619 | Down | C_16_H_32_O_2_ | [M]+ |
| Genistein | 1.1169 | 0.2813 | 270.1696 | Down | C_15_H_10_O_5_ | [M]- |
| N2-gamma-Glutamylglutamine | 1.5031 | 0.8277 | 274.1025 | Down | C_10_H_17_N_3_O_6_ | [M-H]- |
| Oleic acid | 1.2504 | 0.6443 | 282.2795 | Down | C_18_H_34_O_2_ | [M]+ |
| (6Z)-Octadecenoic acid | 1.6644 | 0.5983 | 282.2800 | Down | C_18_H_34_O_2_ | [M]+ |
| 3-Methylxanthine | 1.1633 | 0.8198 | 149.0456 | Down | C_6_H_6_N_4_O_2_ | [M+H-H_2_O]+ |
| Octadecanamide | 1.1217 | 0.6210 | 284.2957 | Down | C_18_H_37_NO | [M+H]+ |
| Hexadecanedioate | 1.0508 | 0.8205 | 285.2047 | Down | C_16_H_30_O_4_ | [M-H]- |
| Indoleglycerol phosphate | 1.8360 | 0.4102 | 288.0749 | Down | C_11_H_14_NO_6_P | [M+H]+ |
| 9,10-Dihydroxystearate | 1.8181 | 0.4167 | 299.2587 | Down | C_18_H_36_O_4_ | [M+H-H_2_O]+ |
| Sphinganine | 1.1597 | 0.7519 | 302.3054 | Down | C_18_H_39_NO_2_ | [M+H]+ |
| Ribose 1,5-bisphosphate | 2.0143 | 0.6099 | 309.1713 | Down | C_5_H_12_O_11_P_2_ | [M-H]- |
| Pergolide | 1.6957 | 0.1837 | 314.1839 | Down | C_19_H_26_N_2_S | [M]+ |
| 1-Arachidonoylglycerol | 1.3190 | 0.5514 | 361.2747 | Down | C_23_H_38_O_4_ | [M+H-H_2_O]+ |
| O-Acetylcarnitine | 1.5024 | 0.3501 | 205.1262 | Down | C_9_H_18_NO_4_ | [M+H]+ |
| Myriocin | 1.4851 | 0.5475 | 402.2860 | Down | C_21_H_39_NO_6_ | [M+H]+ |
| 3beta,5beta-Ketodiol | 1.1046 | 0.7803 | 417.3359 | Down | C_27_H_44_O_3_ | [M+H]+ |
| Lanosterin | 1.3166 | 0.7935 | 425.2539 | Down | C_30_H_50_O | [M-H]- |
| Galactosylsphingosine | 1.6828 | 0.4198 | 461.3255 | Down | C_24_H_47_NO_7_ | [M]+ |
| 3-Epiecdysone | 1.4271 | 0.3086 | 464.2840 | Down | C_27_H_44_O_6_ | [M]+ |
| L-Olivosyl-oleandolide | 2.2581 | 0.6106 | 517.3112 | Down | C_26_H_44_O_10_ | [M+H]+ |
| L-Oleandrosyl-oleandolide | 1.5910 | 0.6045 | 530.3212 | Down | C_27_H_46_O_10_ | [M]+ |
| Avermectin A1b aglycone | 1.1084 | 0.7072 | 567.3269 | Down | C_34_H_48_O_8_ | [M+H-H_2_O]+ |

## Supplementary Figures


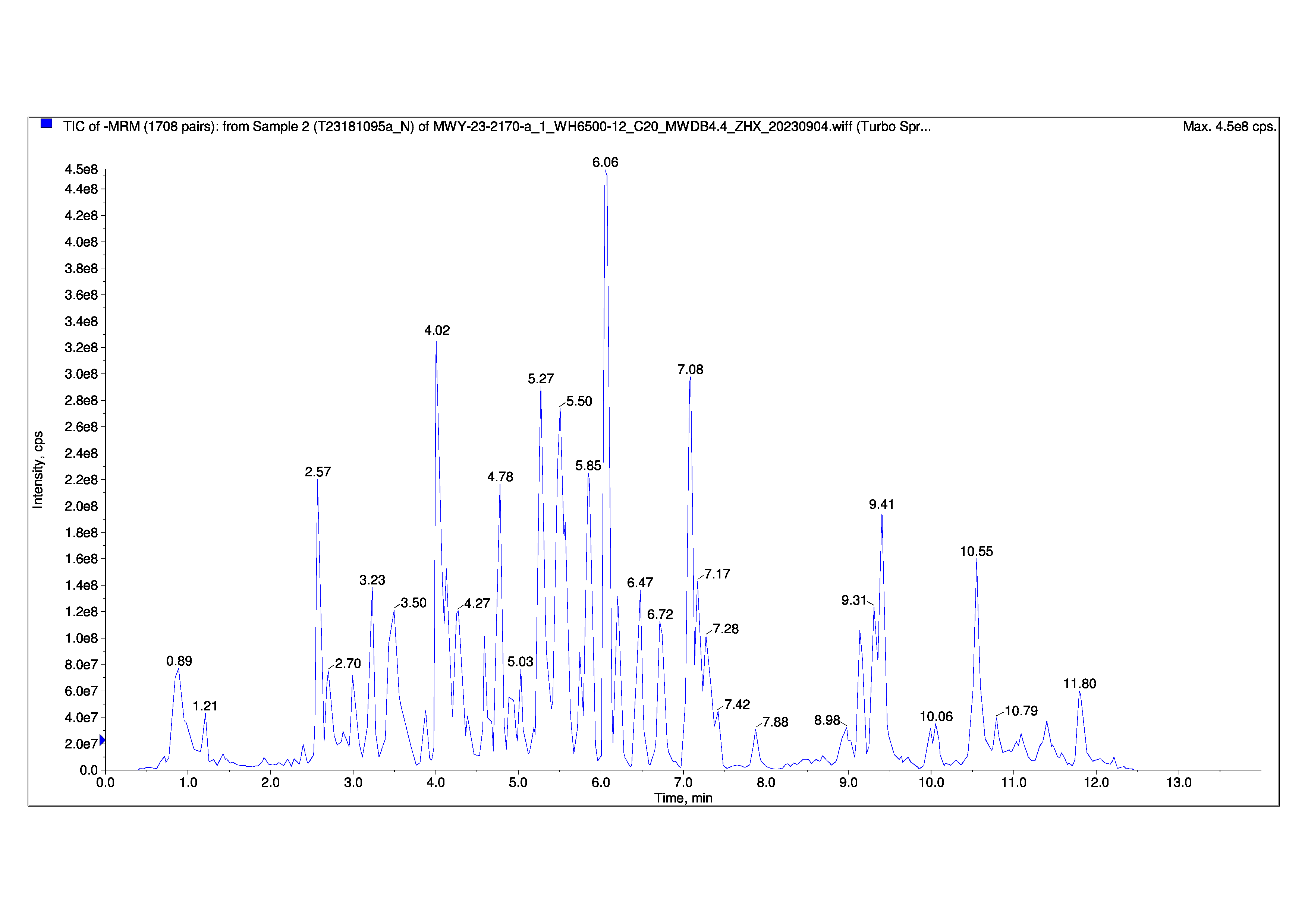

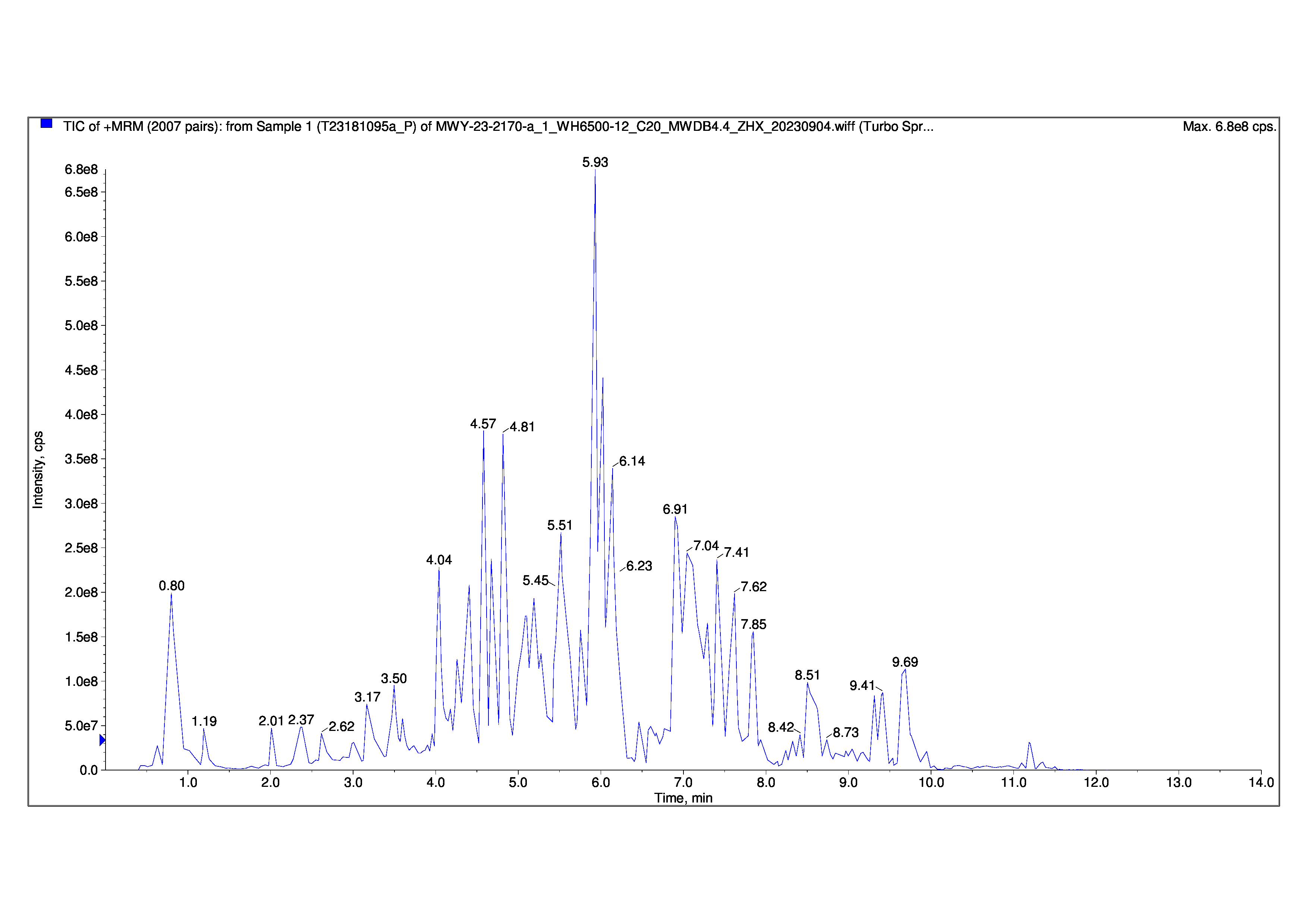


**Supplementary Figure 1** The total ion current chromatogram of the propolis ethanol extract
